# Synthesis and preclinical validation of novel P2Y1 receptor ligands as a potent anti-prostate cancer agent

**DOI:** 10.1038/s41598-019-55194-8

**Published:** 2019-12-12

**Authors:** Hien Thi Thu Le, Tatu Rimpilainen, Saravanan Konda Mani, Akshaya Murugesan, Olli Yli-Harja, Nuno R. Candeias, Meenakshisundaram Kandhavelu

**Affiliations:** 1 Molecular Signaling Lab, Faculty of Medicine and Health Technology, Tampere University and BioMediTech, P.O.Box 553, 33101 Tampere, Finland; 2 Faculty of Engineering and Natural Sciences, Tampere University, Korkeakoulunkatu 8, 33101 Tampere, Finland; 3 Department of Crystallography & Biophysics, University of Madras, Guindy Campus, Chennai, 600025 India; 4 Department of Biotechnology, Lady Doak College, Thallakulam, Madurai, 625002 India; 5 Computational Systems Biology Research Group, Faculty of Medicine and Health Technology and BioMediTech, Tampere University, P.O.Box 553, 33101 Tampere, Finland; 6 Institute for Systems Biology, 1441 N 34th Street, Seattle, WA 98103-8904 USA

**Keywords:** Computational models, High-throughput screening, Receptor pharmacology, Structure-based drug design, Targeted therapies

## Abstract

Purinergic receptor is a potential drug target for neuropathic pain, Alzheimer disease, and prostate cancer. Focusing on the structure-based ligand discovery, docking analysis on the crystal structure of P2Y_1_ receptor (P2Y_1_R) with 923 derivatives of 1-indolinoalkyl 2-phenolic compound is performed to understand the molecular insights of the receptor. The structural model identified the top novel ligands, 426 (compound **1**) and 636 (compound **2**) having highest binding affinity with the docking score of −7.38 and −6.92. We have reported the interaction efficacy and the dynamics of P2Y_1_R protein with the ligands. The best hits synthesized were experimentally optimized as a potent P2Y_1_ agonists. These ligands exhibits anti-proliferative effect against the PC-3 and DU-145 cells (IC_50_ = 15 µM – 33 µM) with significant increase in the calcium level in dose- and time-dependent manner. Moreover, the activation of P2Y_1_R induced the apoptosis via Capase3/7 and ROS signaling pathway. Thus it is evidenced that the newly synthesized ligands, as a P2Y_1_R agonists could potentially act as a therapeutic drug for treating prostate cancer.

## Introduction

Prostate cancer (PCa) is the most common cause of cancer deaths in men^1^. It has been characterized as a complex disease induced by the alteration in intrinsic and extrinsic cellular processes^2^. G-protein coupled receptors (GPCRs), the largest family of cell surface receptor plays a key role in metastatic cancer and hence considered as the promising targets for cancer treatment^3,4^. However, the substantial role of GPCRs in cancer progression and treatment remains questionable. Purinergic receptors (P2YRs), another member of GPCR family, found to be over-expressed in some types of cancer cells and tissues^5^. Based on the differences in gene sequence, protein structure, and functions, the P2YR family constitutes 8 homo-receptor subtypes, such as P2Y_1_, P2Y_2_, P2Y_4_, P2Y_6_, P2Y_11_, P2Y_12_, P2Y_13_, and P2Y_14_^6^. Of late, it has been identified that P2Y_1_ expression is higher in PC-3^7–9^ and DU-145 cells^9,10^ both in normal and stimulation condition than in non-cancerous cells. Therefore, P2Y1R is considered as a noteworthy tumor cell marker and anticipated to be used as a target for inhibiting the PCa cell proliferation.

The stimulations of P2Y_1_R induce corresponding signal transduction pathways that varied for different cell types^11^. The selected P2Y1R-targeted agonist, MRS 2365 increases lactate dehydrogenase and intracellular calcium (Ca^2+^) levels, in turn induces apoptosis and inhibits the PC-3 cells proliferation^8^. Furthermore, 2-MeSADP, a non-selective P2Y_1_ agonist, stimulates intracellular Ca^2+^, cell death and reduces cell aggression in 1321N1 astrocytoma cells transfected with the human P2Y_1_R^12,13^. Still, in HUVEC cells, a P2Y_1_R antagonist MRS2179 leads to the formation of phosphatydilinositol, and phosphorylates the mitogen-activated protein kinases (MAPK)^14,15^. The activation of MAPK signaling possibly contributes to the re-endothelialization after vascular injury^14,15^.

Other potential therapeutic applications for P2Y_1_R ligands includes, agonist as antidiabetic agents or antagonists as antithrombotic agents *in vitro* and *in vivo* models^16–18^. Although there is expression of P2Y_1_R in the human prostate, its role in the growth of PCa is yet to be characterized. In the present study, PC-3 and DU-145 PCa cells^19,20^, were used to investigate the effect of P2Y_1_R and novel agonists in cell death and proliferation. Many scaffolds such as 1,4-substituted triazoles, pyrimidines or pyrazoles are known for their antitumor activities^21–23^. Similarly, phenolic Mannich bases were recognized to possess anticancer and cytotoxic activity^24^. Derivatives of aminomethylated naphthols and 8-hydroxyquinoline induces apoptosis on activation of caspase-dependent pathways^24,25^.

Our earlier reports have also demonstrated the ability of 1-indolinoalkyl 2-phenols to inhibit cancer cells growth^26^. Since phenolic compounds have profound role in inhibiting the cancer cell proliferation, a large variety of substituents of 1-indolinoalkyl 2-phenols is considered in the initial library for the docking studies. We synthesized a group of 1-indolinoalkyl 2-phenolic derivatives using 3-component Petasis borono-Mannich reaction (i.e., salicylaldehydes, indolines and boronic acids) and many potential hits are experimentally verified. Based on the probability of targeted P2Y_1_R signaling activation to inhibit PCa cell growth, a library of over 900 structures was built with single variation in the substituents from the different components along with their combinations. The best docking poses in the ligands interaction with P2Y_1_R was further analyzed. The detailed interaction of the three-dimensional structure of P2Y_1_R with the selective antagonist MRS2179 was performed for scrutinizing the newly synthesized ligands. The competence of new P2Y_1_ ligand identified via molecular modeling, docking, and calcium kinetics is analyzed. The activation of P2Y_1_R down-stream signaling pathway and their effect in PCa is also explored through apoptosis, ROS and Caspase 3/7 assays. Our findings suggested that the identified ligands might potentially help in the treatment of the prostate cancer.

## Results and Discussion

### Novel ligands of P2Y_1_R

**Figure 1:**
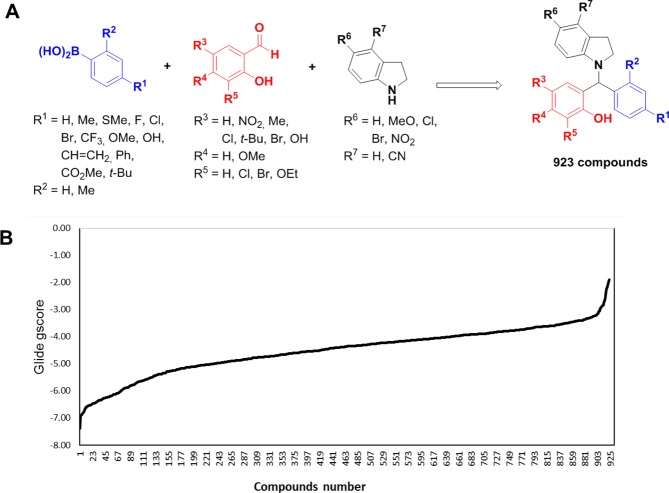
Hit identification based on docking score. (**A**) Library of 1-indolinoalkyl 2-phenols for P2Y_1_R docking screen. (**B**) Glide docking score (kcal/mol) of 923 compounds against P2Y_1_R. Two ligand-like compounds **1** and **2** ranks top with high docking scores.

The three – dimensional (3D) coordinates of P2Y_1_R was retrieved from Protein Data Bank with the code 4XNW (Resolution: 2.7 Å) comprising of 427 amino acid residues. The P2Y_1_ protein model shares a canonical seven transmembrane helices each flanked by the topological domain like other known GPCR structures^27,28^. To study the binding mode of P2Y_1_R, initially we performed the docking studies with the known antagonist MRS2500 (co-crystallized)^29^, and an agonist MRS2365 (glide score −8.80 Kcal/mol) using Schrodinger. Over 900 compounds were designed using Java Molecular Editor (JME) and translated to structure data file which is compiled in the repository (Table S2 of SI) (Fig. 1A). P2Y_1_R model was docked with 923 compounds (Fig. 1B and Table S2 of SI). The docked results were analyzed based on the presence of hydrogen bonds, salt bridges, halogen bonds, aromatic bonds, π-cation and π-π interactions. All the conformers were scrutinized based on the binding mode and the stability of the protein-ligand complex. The library comprising 923 compounds was screened based on the docking score that are −7.0 and above (Fig. 1B and Table S2 of SI). The best two ligand like compounds 1 and 2 with the highest docking score, that satisfies Lipinski’s rule were selected. The high glide score indicated a high binding affinity towards the P2Y_1_R.

**Figure 2:**
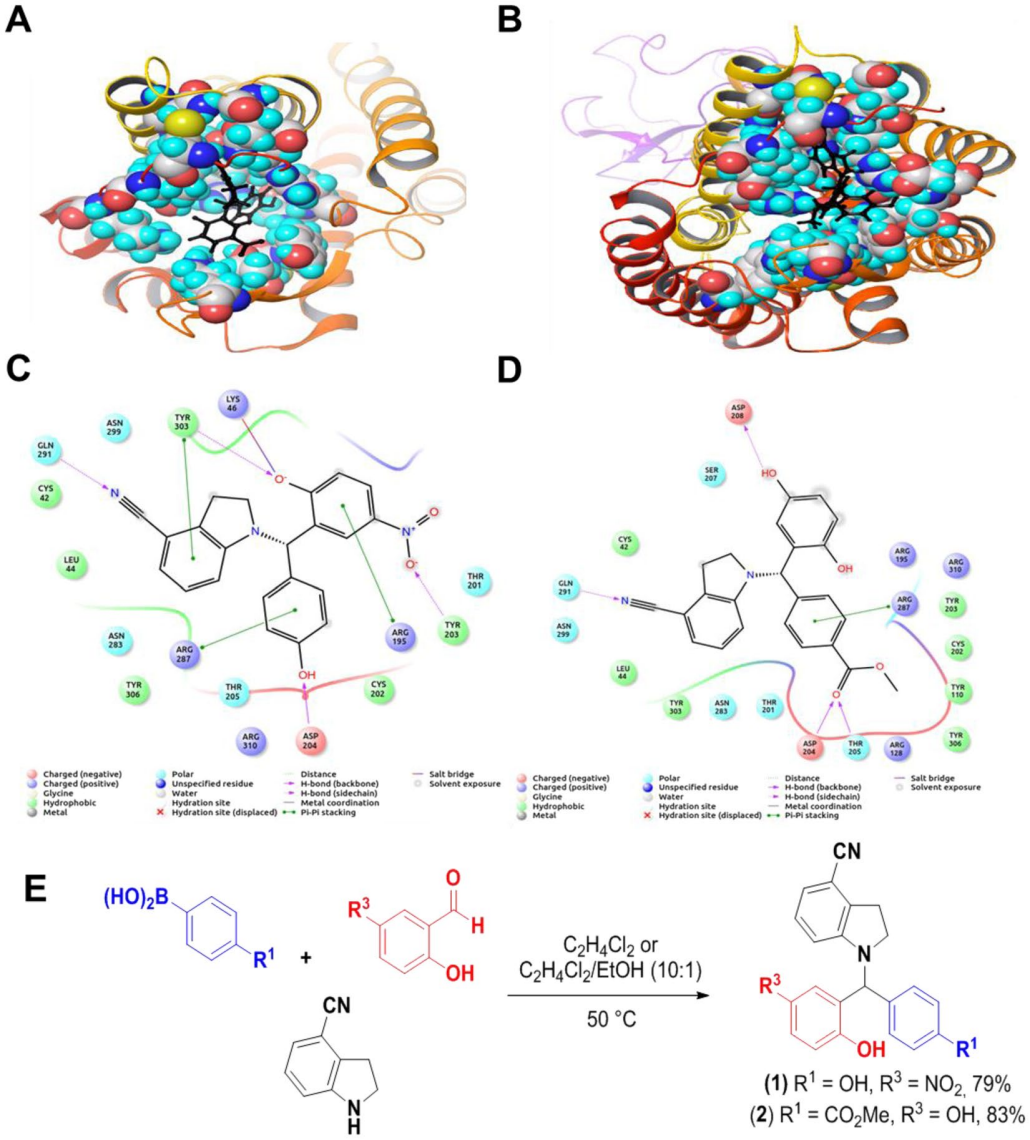
The ligand binding residues of the receptor is shown as the surface model and the ligand is shown in black colored ball and stick model (**A**) compound 1 and P2Y1 (**B**) compound 2 and P2Y1. The non-ligand interacting regions of receptor is shown as ribbon model. (**C**) Two-dimensional ligand interaction diagram of compound **1** and (**D**) compound **2**. The color coding and interactions are described in the ligand key. (**E**) Synthesis scheme of 1-indolinoalkyl 2-phenols **1** and **2**.

2D ligand interaction diagram showed the similar number of interaction of ligands with amino acid residues in the P2Y_1_R, **1** with 16 interactions (Fig. 2A) and 2 with 19 interactions (Fig. 2B). Six hydrophobic interactions were found between **1** and P2Y_1_R while seven interactions between **2** and the receptor. Both ligands form interaction with cysteine, tyrosine and sulfur containing amino acid residues of the P2Y_1_R. The Hydrophobic contact between the protein and ligands are the key property for the protein folding and stability^30^. The charged residue interactions were also observed between ligands and P2Y_1_R molecule at Arg287. Cation-pi stabilizing electrostatic interactions were found in similar number in both the ligands (Fig. 2C,D). There are a few interactions found to be conserved on both P2Y_1_R-ligand **1** and **2** complexes at the amino acid residues such as Arg287, Arg310, Arg195 (Charged^38^), Tyr303, Cys42, Cys202 (Hydrophobic), and Leu44. The presence of cysteine residues at the interface is also essential for maintaining the precise pocket formation that allows the receptor to bind with the ligands^30^. These observations suggest that both ligands have the potentiality to bind with P2Y_1_R.

The identified promising hits were organized through the abovementioned Petasis borono-Mannich reaction (Fig. 2E). Indoline-4-carbonitrile was prepared with 68% yield on reducing the corresponding indole with triethylsilane in TFA^31^. Both 1-indolinoalkyl 2-phenols was obtained in good yields upon reaction at 50 °C, while preparation of **2** requires longer reaction time (20 h vs 70 min for **1**) due to the lower reactivity of the boronic acid partner.

### Novel ligand-P2Y1R interaction and signaling activation affects intracellular calcium

**Figure 3:**
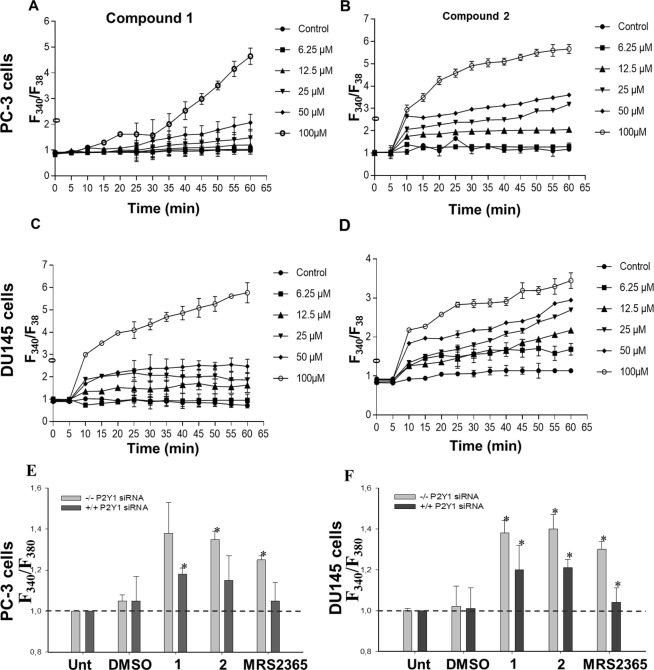
Measurement of intracellular calcium with Fura 2-AM on activation of P2Y1R by compound **1** and **2**. The fluorescence was measured using Magelan^TM^ microplate plate reader at every 5 min. The ratiometric Ca^2+^ fold change was analyzed based on the emitted fluorescence intensities of the samples. PC-3 cells were treated with (**A**) compound **1**, (**B**) compound **2** and DU-145 cells with (**C**) compound **1** and (**D**) compound **2**. (**E**) P2Y1R silencing by siRNA and and its effect on Ca^2+^ signaling activation by compound **1**, **2** and MRS2365 in PC-3 cells (**F**) same condition as “E” in DU-145 cells. The experiments were repeated 3 independent times, *p < 0.05 by one-way ANOVA.

The activation of phospholipase C (PLC) is the common signal transduction pathway triggered by the P2Y_1_R-G_q_^32^. Phosphatatidylinositol-4,5-bisphosphatase is hydrolyzed by the PLC activation, which increases the cytosolic Ca^2+^ mobilization through the generated IP3 and diacylglycerol^33^. To elucidate the agonistic activity of these two compounds, we analyzed the changes in the downstream effector, Ca^2+^ in PCa cells^34^. As shown in Fig. 3, intracellular Ca^2+^ concentration increases in PC-3 (Fig. 3A,B) and DU-145 (Fig. 3C,D) cells over the concentration of compound **1** and **2** in a time dependent manner. As evident from Fig. 3A,C, compound **1** at 100 µM concentration increased the Ca^2+^ level in PC-3 and DU-145 cells which is 5 fold higher than the untreated condition after 60 min. Similarly, compound **2** at 25 µM also increased the Ca^2+^ by 3 fold higher than the untreated condition after 60 min. siRNA assay was also performed to confirm the P2Y_1_R targeted binding of the compound **1** and **2**. In the absence of P2Y_1_ siRNA, there was 1.3 fold higher level of Ca^2+^ upon the activation of P2Y_1_R signal by MRS2365, compound **1** and **2**, whereas the presence of P2Y_1_ siRNA showed 0.2 fold decrease in the level of Ca^2+^ in PC-3 cells (Fig. 3E) and DU- 145 cells (Fig. 3F). These results shows the P2Y_1_R specific interaction of the novel ligands that can act as an agonist which is congruent with the virtual screening results.

### Growth inhibitory effects of compound 1 and 2 on PC-3 and DU-145 cells

**Figure 4:**
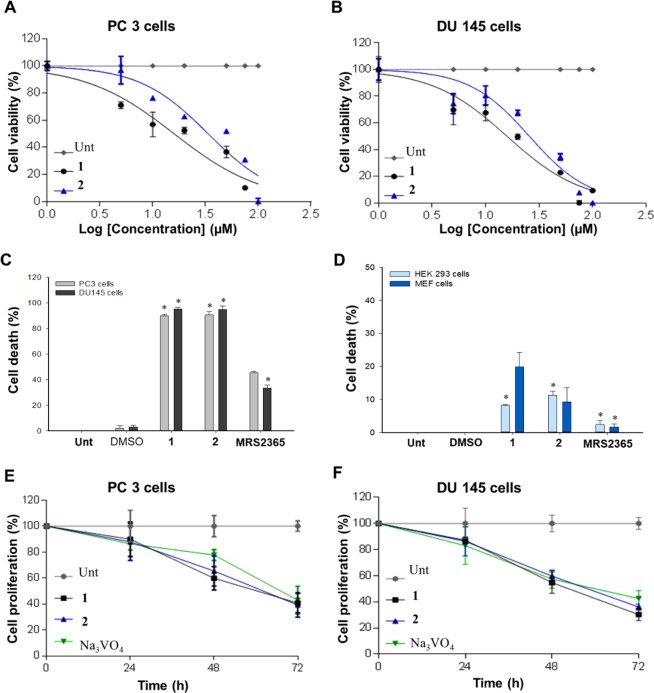
Effect of compound **1** and **2** on the cell viability. (**A**) PC-3 cells and (**B**) DU-145 cells were treated with the varying concentrations of compound **1** and **2**. IC_50_ of the untreated cells along with the respective compounds were determined by Prism 7.0. (**C**) PC3 and DU-145 cells and (**D**) HEK293 and MEF cells were treated with 100 µM of compound **1** and **2**, MRS2365 for 48 h with DMSO as negative control. (**E**) PC-3 and (**F**) DU-145 cells were incubated with the IC_50_ concentration of compound **1** and **2** and 50 µM Na_3_VO_4_ for 24 h, 48 h, and 72 h. The experiment was performed with replicates of biological and technical repeats. Statistical significance was considered at *p < 0.05.

P2Y_1_R has been used as a biomarker for the therapeutic treatments of PCa cells^35,36^ and its agonists acting as an cell death inducer^9,12^. In the present study, compound **1** and **2** were chosen as an ideal ligand based on its potentiality to bind and interact with the P2Y_1_R. Further to explore the cytotoxicity effect of these two ligands against the growth of PCa cells, MTT assay was performed. PC-3 and DU-145 cells were treated with varying concentrations of compound **1** and **2**. As given in the Fig. 4A, the compounds decreased the cell viability when relatively compared with the untreated control group. Dose-dependent experiment on PC-3 cells revealed the IC_50_ values as 15.98 µM for compound **1** and 33.57 µM for compound **2**. Apparently, IC_50_ values for DU-145 cells was found to be 15.64 µM for compound **1** and 25.64 µM for compound **2** (Fig. 4B). Based on the IC_50_ values, compound **1** exerted a better cytotoxic effect on PCa cells than compound **2**. Notably, compound **1** and **2** induced ~96% of cell death in PCa cells whereas MRS2365, the positive control of P2Y_1_R agonist, induced about 38% of cell death (Fig. 4C). In contrast, HEK293 and MEF, non-cancerous cells, were significantly less sensitive when treated with compound **1**, **2**, and MRS365 than the PCa cells (Fig. 4D). The cell death of non-cancerous cells was observed to be less than 20% with 100 μM concentration of compound **1** and **2** treatment. These observation concluded that the compound **1** and **2** have cytotoxic effect specific for PCa cells.

To detect the effect of the compounds, the PC-3 and DU-145 cells were treated at different time points with IC_50_ concentration of **1** and **2**. Figure 4E,F have shown that the cell proliferation in the treated cells were significantly lower than the control group over the time. The effect of compound **1** and **2** on PC-3 reduced the cell proliferation to about 89%, 67%, and 42% and 90%, 69%, 40% respectively at 24 h, 48 h, and 72 h. Similarly, the impact of Na_3_VO_4_ on PC-3 cells have shown the reduced proliferation of about 92%, 81%, and 45% at 24 h, 48 h, and 72 h, respectively. The inhibition of cell proliferation of the compound **1** and **2** was higher than the positive control Na_3_VO_4_ at 48 h (Fig. 4E). In contrast, DU-145 cells, the cell growth was reduced from ~85% to ~60% progressively from 24 h to 48 h on treatment with compound **1**, **2** and Na_3_VO_4_ (Fig. 4F). However, at 72 h, the proliferation was inhibited to 43% on treatment with compound **1** and **2**, which was higher than Na_3_VO_4_ treatment. These findings supports the hypothesis that compound **1** and **2** could inhibit the cancer cell proliferation on increasing the treatment time.

### Effects of compound 1 and 2 on PCa apoptosis

**Figure 5:**
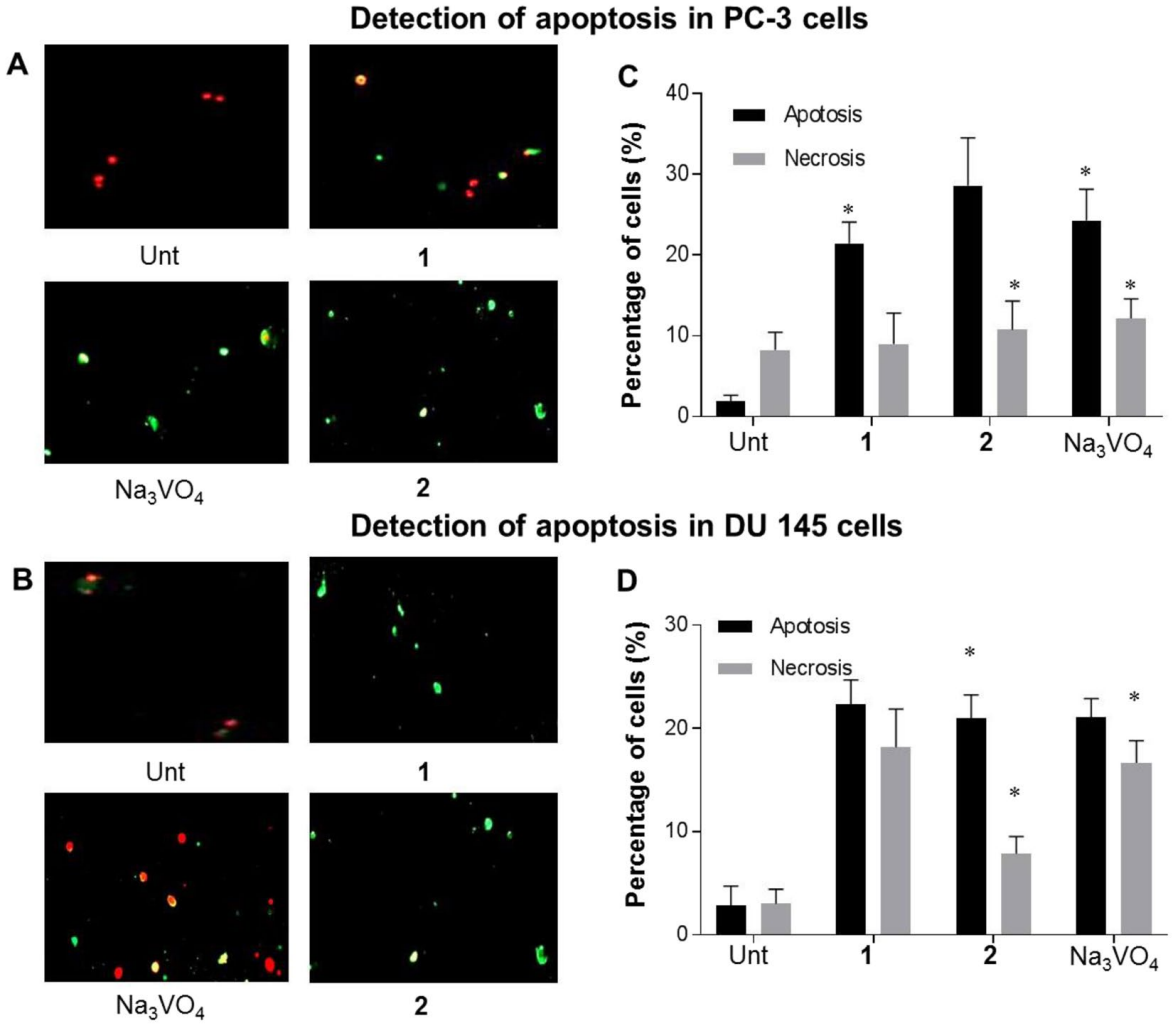
Induction of apoptosis by compound **1** and **2** on PCa cells. (**A**) Representative images of PC-3 cells stained with Annexin-V/PI in untreated, compound **1**, **2**, and Na_3_VO_4_ treated condition. (**B**) percentage of apoptotic and necrotic cell death in the corresponding condition as in A. (**C**) The representative images of DU-145 cells stained with Annexin-V/PI (**D**) the percentage of apoptosis and necrosis in DU-145 cells. Results are represented as mean of three independent experiments, mean ± S.D. *P < 0.05, versus control, n = 3.

Apoptosis is a common response to cell stress during the process of cell death^37^ which can happen through the increase of intracellular Ca^2+^, ROS and the activation of caspase^38^. We sought to determine the efficacy of the novel agonists **1** and **2** on PCa cell lines, Annexin V-affinity assay was performed. After 48 h of treatment, the fluorescent microscope images of PC-3 (Fig. 5A) and DU-145 cells (Fig. 5B) exposed the presence of apoptotic and necrotic cells. PC-3 cells on treatment with compound **1** and **2** caused apoptosis of 23.2% and 29.6% whereas the positive control Na_3_VO_4_ caused 25.6% apoptosis (Fig. 5C). DU-145 cells after 48 h treatment with compound **1**, **2** and Na_3_VO_4_ is marked with 20% increase in apoptotic cell fraction when compared to the untreated cells (Fig. 5D). Taken together, we conclude that the activation of P2Y_1_R by the compound **1** and **2** increases the cell death through apoptosis.

### Production of ROS by compound 1 and 2 in PCa cells

**Figure 6:**
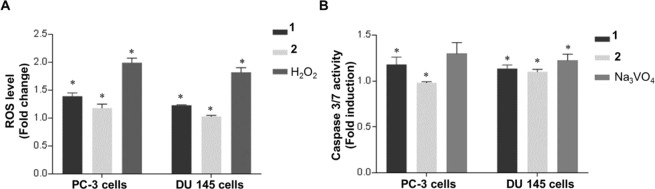
Production of ROS and activation of Caspase3/7 by compound **1** and **2** in PCa cells. (**A**) The fold change in ROS in PC-3 and DU-145 cells treated with compound **1**, **2** and H_2_O_2_. H2DCFDA labelled cells was used to measure the ROS production and its fluorescence signal was recorded using 96 well plate reader. The fold change of ROS was calculated using fluorescence intensities of the untreated control. (**B**) The fold change in Caspase 3/7 in PC-3 and DU-145 cells treated with compound **1**, **2**, and Na_3_VO_4_. Biological and the technical replicates were maintained to assess the significance of the results, with mean ± S.D. *P < 0.05, versus control, n = 3.

ROS production exists under normal and abnormal physiological conditions of the cell^39^. The production of ROS affects several signaling pathways such as cell survival, phosphatase and kinase activities, and muscle plasticity^40^. ROS promotes many events of tumor progression like cell proliferation, metastasis, and angiogenesis^41^. ROS is also capable of inducing cell cycle arrest and cell death in cancer treatment^42^. In order to explore the effect of P2Y_1_R activation on prostate cancer via ROS, PC-3 and DU-145 cells were incubated with compound **1**, **2**, and H_2_O_2_. As shown in Fig. 6, the production of ROS increased in the presence of H_2_O_2_, compound **1** and **2** in PC-3 and DU-145 cells (Fig. 6A). We noticed an increase in the fold change of ROS to 1.41 and 1.22 in the compound **1** and **2** treated PC-3 cells respectively, when compared to the untreated condition. The positive control H_2_O_2_ expressed 2.1 fold change in ROS level which is greater than the compound **1** and **2** treatment. Likewise, ROS level in DU-145 cells also increased to 1.36 and 1.01 fold change on treatment with compound **1** and **2** whereas H_2_O_2_ showed 1.78 ROS fold change. The difference in the fold change was proven to be statistically significant by ANOVA test with the P*-value < 0.05* (Table S1 of SI). These results indicates that the agonists **1** and **2** enhanced the production of ROS in both PCa cells.

### Activation of Caspase 3/7 by compound 1 and 2 on PCa

Apoptosis is induced through the activation of intracellular caspases and lead to the modification of protein substrate within the nucleus and cytoplasm^43^. Currently more than 14 caspases were cloned and partially their functions were determinuteed to be in programmed cell death^44^. Among them, caspases 3 and 7 have been identified as an executioner caspases that directly lead to the intrinsic/extrinsic pathways in apoptosis process^45,46^. Since the caspase plays an essential role in cell death, the anti-cancer effect of agonist **1** and **2** were explored by determinuteing the changes in the caspase 3/7 activity. As described in the Caspase-Glo® 3/7 assay, PC-3 and DU-145 cells were treated with compounds **1**, **2**, and Na_3_VO_4_. Interestingly, PC-3 cells treated with compound **1** exhibited an increase of caspase 3/7, showing 1.22 fold induction when compared to the untreated cells (Fig. 6B). Besides, compound **2** and positive control exhibited 0.8 and 1.26 fold induction, respectively. However, Caspase 3/7 activity increased similarly around 1.15-fold change in DU-145 cells on treatment with compound **1** and **2** than the untreated condition. The difference in the fold change of treated and untreated conditions were statistically significant as per ANOVA test (P-value < 0.05; Table S1 of SI). Collectively, the results demonstrated that the novel agonist **1** and **2** could induce apoptosis through Caspase 3/7 dependent signaling pathway.

## Conclusion

P2Y_1_R, a purinergic G_q_ protein, has been reported as the pharmacological target for the therapeutic treatment of PCa^20,47,48^. In the present research, molecular docking experiments was performed to investigate the interaction of a library of 923 1-indolinoalkyl 2-phenolic derivatives with P2Y_1_R protein. Docking analysis revealed that the compound **1** and **2** as the novel ligands. Furthermore, interactions of P2Y_1_R between these two ligands demonstrated the crucial aminuteo acid interactions responsible for the folding and stability. The synthesized 1-indolinoalkyl 2-phenolic derivatives **1** and **2** were purified and used for the activation of P2Y_1_R, resulted in the increase of intracellular Ca^2+^ in PCa cell. The compound **1** and **2** induced Ca^2+^ level in a dose/time-dependent manner suggesting that these compounds are agonists for P2Y_1_R. In addition, the activation of P2Y_1_R induced the cell death with IC_50_ concentration of 15–33 µM. The compound **1** and **2** promoted apoptosis and necrosis which increased ROS production and caspase 3/7 signaling. These results demonstrated that the findings are consistent with the earlier reports on the functional effect of P2Y_1_R activation in PCa cells^8,49^. We suggest that P2Y_1_R might be an attractive target for the treatment of prostate cancer. Thus it is concluded that the synthesized 1-indolinoalkyl 2-phenolic derivatives **1** and **2** could provide the new opportunity to develop P2Y_1_-signaling mediated drugs for the treatment of PCa.

## Materials and Methods

### Structure model

Structure of the P2Y1R was retrieved from PDB with the identification code 4XNW^49^. The crystal structure of the human P2Y1R in complexed with the nucleotide antagonist MRS2500 at 2.7 Å resolution is used as a reference compound. Protein Preparation Wizard in Maestro^50^ is used for the preparation of the 3D structure of the protein. Protein structure was stabilized by adding and optimizing the hydrogen atoms and bonds, removing atomic clashes, adding formal charges to the hetero groups and then optimizing at neutral pH. Finally, the structure was minuteimized with optimized potential for liquid simulations force field (OPLS-2005). The ligand binding site observed in the crystal structure is used as the control binding site whereas, docked complex with the known agonist, MRS2365 is used as the positive control. This is used to perform the further docking of 923 conformers.

### Ligand library

The two-dimensional structures of 923 aminuteobenzylated phenols were generated using RD Kit library for Python and exported to Structure Data File (SDF). The ligand molecules were subjected to LigPrep module of Schrödinger suite^51^. This module is used to generate the possible low energy stereoisomers with standard physical conditions. The prepared 923 ligands were subjected to high throughput virtual screening using the GLIDE (Grid based Ligand and Docking with Energetics) module of Schrödinger suite^52^.

### Docking screening

Receptor grid box for the 923 compounds were generated using the ligand binding site of the crystal structure (P2Y1R complexed with MRS2500). Ligands were docked to the protein using Glide software. Docking was performed in a “Standard Precision” (SP) mode and then by “Extra precision” mode (XP). The docked conformers were evaluated using Glide (G) Score^53^.

### Design and synthesis of P2Y_1_ ligands with general remarks

The reactions were performed using the reagents from Sigma-Aldrich or TCI, and the experiment was performed under argon atmosphere. Thin-layer chromatography was done on pre-coated (Merck TLC silica gel 60 F254) aluminuteium plates, developed using cerium molybdate solution and visualized under UV light. Flash column chromatography was done on silica gel 60 (Merck, 0.040–0.063 mm). NMR spectra were recorded (Jeol ECZR 500) using CDCl_3_ as solvent and calibration was done using tetramethylsilane as internal standard. Chemical shifts in ppm (δ) are specified to the CDCl_3_ residual peak (δ 7.26) or TMS peak (δ 0.00) for ^1^H NMR, to CDCl_3_ (δ 77.16) for ^13^C NMR. The peak splitting patterns were designated as; s = singlet, d = doublet, t = triplet, m = multiplet. Coupling constants, *J*, is represented in Hertz (Hz). High-resolution mass spectra was recorded on the Waters ESI-TOF MS spectrometer. Elemental analysis to detect C, N and H was determinuteed on Elementar vario EL III. Tested compounds shows purity > 95% upon elemental analysis. Indoline-4-carbonitrile was prepared as the earlier method for reducing the corresponding indole with triethylsilane^31^ with the same spectral characterization^54^ (Fig. S1 of SI).

### 1-(2-Hydroxy-5-nitrophenyl)(4-hydroxyphenyl)methyl)indoline-4-carbonitrile (1)

Indoline-4-carbonitrile (71 mg, 0.5 mmol) was added to 2-hydroxy-5-nitrobenzaldehyde (84 mg, 0.5 mmol, 1 equiv) and (4-hydroxyphenyl) boronic acid (69 mg, 0.5 mmol, 1 equiv) in 5.0 mL DCE and 0.5 mL EtOH at 50 °C. The reaction was stirred for 70 minuteutes and the solvents were evaporated under reduced pressure. The gradient column chromatography was to purify the residue (DCM to DCM/EtOAc 85:15) to give compound **1** (152.7 mg, 0.39 mmol, 79% yield) as a light yellow solid. ^1^H NMR (500 MHz, CDCl_3_) δ 10.42 (br. s, 1 H), 8.12 (dd, *J* = 9.2, 2.9 Hz, 1 H), 7.98 (d, *J* = 2.9 Hz, 1 H), 7.24 (d, *J* = 8.6 Hz, 2 H), 7.12–7.06 (m, 2 H), 6.97 (d, *J* = 9.2 Hz, 1 H), 6.84 (d, *J* = 8.6 Hz, 2 H), 6.60 (d, *J* = 8.0 Hz, 1 H), 5.55 (br. s, 1 H), 5.38 (s, 1 H), 3.33 (td, *J* = 8.7, 3.8 Hz, 1 H), 3.25–3.07 (m, 3 H). ^13^C NMR (126 MHz, CDCl_3_) δ 161.9, 156.4, 151.1, 141.2, 136.9, 130.3, 129.3, 128.8, 126.6, 125.5, 125.0, 124.5, 117.8, 117.3, 116.3, 115.6, 109.2, 68.3, 52.9, 28.1. Elemental analysis: Calcd for C_22_H_17_N_3_O_4_: C, 68.21; H, 4.42; N, 10.85. Found: C, 65.03; H, 4.47; N, 9.81. HRMS (ESI/TOF): m/z calcd for C_22_H_16_N_3_O_4_^−^ [M − H]^−^, 386.1146; found 386.1129 (Fig. S2 and S3 of SI).

### Methyl 4-((4-cyanoindolin-1-yl)(2,5-dihydroxyphenyl)methyl)benzoate (2)

Indoline-4-carbonitrile (71 mg, 0.5 mmol) was added to a solution of 2,5-dihydroxybenzaldehyde (69 mg, 0.5 mmol, 1 equiv) and (4-(methoxycarbonyl)phenyl)boronic acid (90 mg, 0.5 mmol, 1 equiv) in 5.0 mL DCE at 50 °C. The reaction was agitated continuously for 20 h and the solvent were evaporated under reduced pressure. The residue was purified (gradient column chromatography, hexane/iPrOH 85:15 to hexane/iPrOH 80:20) to produce compound **2** (165.3 mg, 0.41 mmol, 83% yield) as an off-white solid. ^1^H NMR (500 MHz, CDCl_3_) δ 7.94 (d, *J* = 8.0 Hz, 2 H), 7.40 (d, *J* = 8.6 Hz, 2 H), 7.31 (br. s, 1 H), 6.95 (t, *J = *7.7 Hz, 1 H), 6.90 (d, *J* = 7.4 Hz, 1 H), 6.73 (d, *J* = 8.6 Hz, 1 H), 6.66 (dd, *J* = 8.6, 2.9 Hz, 1 H), 6.52 (d, *J* = 2.9 Hz, 1 H), 6.43 (d, *J* = 8.0 Hz, 1 H), 5.89 (br. s, 1 H), 5.62 (s, 1 H), 3.87 (s, 3 H), 3.27 (t, *J* = 8.3 Hz, 2 H), 3.13–3.01 (m, 2 H). ^13^C NMR (126 MHz, CDCl_3_) δ 167.2, 151.7, 149.6, 148.3, 144.6, 135.7, 130.2, 129.5, 128.5, 128.5, 126.1, 121.8, 117.7, 117.5, 116.2, 115.7, 113.4, 108.4, 63.9, 52.5, 51.9, 27.9. Elemental analysis: Calcd for C_24_H_20_N_2_O_4_•1.05H_2_O: C, 68.74; H, 5.61; N, 6.68. Found: C, 68.42; H, 4.87; N, 6.60. HRMS (ESI/TOF): m/z calcd for C_24_H_20_N_2_O_4_Cl^−^ [M + Cl]^−^, 435.1117; found 435.1079 (Fig. S4 and S5 of SI).

### Cell culture

PC-3 and DU-145 cells were maintained in Minuteimal Essential Medium Eagle (MEM; Sigma-Aldrich, St. Loius, MO, USA). HEK 293 and MEF cells were maintained in Dulbecco’s modified eagle’s medium. Mediums were supplemented with 10% fetal bovine serum (FBS; Biowest, France) and 1% penicillin/streptomycin (Sigma-Aldrich). Cells were cultured at 37 °C in humidified atmosphere of 5% CO_2_. The media was changed once every 2 days. The culture was passaged using trypsin-EDTA (Sigma-Aldrich). Newly synthesized compounds **1** and **2** were diluted in dimethyl sulfoxide (DMSO, Sigma -Aldrich).

### Cell viability measurement

PC-3, DU-145, HEK 293 and MEF cells were seeded with 1 × 10^4^ cells/well in 96-well plates. At 70–80% confluence, cells were exposed to compound **1**, **2**, DMSO, and MRS2365 for 48 h. MTT and cytotoxicity assay (Bosterbio, CA, USA) was done to check the cell viability, as instructed by the manufacturer and the absorbance was measured at 570 nm using Magellan™ microplate reader (Tecan Group Ltd., Switzerland). Briefly, the cytotoxicity index was determinuteed using the untreated cells as control. DMSO was used as the vehicle control against compound **1** and **2**. The inhibition percentage of each compound, was calculated using the equation given below^55^.

$$ \% \,inhibition=\frac{{A}_{c}\,-{A}_{tr}}{{A}_{c}}\times 100$$

A_c_, cell number of untreated cells; A_t_, cell number of treated cells. A half maximal inhibitory concentration (IC_50_) was determinuteed using the curve fitting program Prism 7.0 (GraphPad Software Inc., La Jolla, CA, USA).

### Cell proliferation assay

96-well plates was seeded with 1 × 10^4^ cells/well concentration of PC-3 and DU-145. The overnight cultured cells were treated with compound **1** and **2** with the IC_50_ concentration or 2 mM sodium orthovanadate (Na_3_VO_4_; positive control)^56^, and maintained in the 5% CO_2_ incubator for 24 h, 48 h, and 72 h. MTT cell proliferation and cytotoxicity assay was performed to measure the cell survival following the manufacturer’s instruction and the absorbance was measured at 570 nm using Magellan^TM^ microplate reader. The cell viability was calculated as the percentage of cell number of treated cells relative to cell number of untreated cells at 24 h, 48 h, and 72 h.

### Calcium kinetic assay

To carry out calcium kinetic assay, PC-3 and DU-145 cells 96-well black plate was plated with 1 × 10^4^ cells/well as previously described^57^. After overnight incubation, the cells were washed with warm 1X phosphate buffered saline (PBS) (pH 7.2). The cells were further incubated with 2 µM Fura 2-AM (Sigma-Aldrich) and 0.01% Pluronic® F-127 (Sigma-Aldrich) in PBS for 30 minute at RT in dark condition. Compound **1** and **2** were prepared in PBS with varying concentration of 6.25 µM, 12.5 µM, 25 µM, 50 µM, and 100 µM. The reaction was started on adding the compounds to the dye and the fluorescence intensity was measured using Magelan^TM^ microplate reader at 37 °C at every 5 minute. The excitation was calculated in two different alternative wavelength 340 nm and 380 nm and the emission of fluorescence was measured at 510 nm. The fold change of intracellular calcium was calculated following the equation below^58^.

$${F}_{340/380}=\frac{{F}_{340}^{tr}-{F}_{340}^{bg}}{{F}_{380}^{tr}-{F}_{380}^{bg}}$$

F_340/380_, fold change of intracellular calcium; ${F}_{340}^{tr}$ Emitted fluorescence intensities of samples with compound at 340/510 nm; ${F}_{380}^{tr}$ Emitted fluorescence intensities of samples with compound at 380/510 nm; ${F}_{340}^{bg}$ Background corrected emitted fluorescence intensities of samples without compound at 340/510 nm; ${F}_{380}^{bg}$ Background corrected emitted fluorescence intensities of samples without compound at 380/510 nm. siRNA assay was also performed to check the specificity of the ligand binding with P2Y_1_R. Predesigned siRNA against human P2Y_1_R was commercially synthesized (cat no. AM16708; Thermo Fisher Scientific, Waltham, MA, USA). PCa cells with the confluence of 60–70% were transfected with 20 nM of siRNA by Lipofectamine RNAiMAX Transfection Reagents (cat no. 13778030; Thermo Fisher Scientific). After 48 h of transfection, cells were measured to quantify the changes in the intracellular calcium level.

### Detection of reactive oxygen species (ROS) formation

12-well plates was seeded with 1 × 10^5^ cells/well of PC-3 and DU-145 cells. After incubation overnight, the cells were treated with compound **1** and **2** for 5 h with their respective IC_50_ concentration or 10 mM hydrogen peroxide (H_2_O_2_), as positive control for ROS^59,60^. The cells were centrifuged at 3,000 rpm for 10 minute and the cell pellets were harvested. Cells were stained with 2 µM molecular probe 2’,7’-dichlorodihydroflurescein diacetate (H_2_DCFDA) for 10 minute in the dark. Subsequently, the stained cells were washed with warm PBS and incubated in the medium for 20 minute. Florescence of ROS was measured at 485 nm and 538 nm using Magelan^TM^ microplate reader. The fold change of ROS product was determinuteed using the equation mentioned below^61^.

$$Fold\,increase=\frac{{F}_{test}-{F}_{blank}}{{F}_{control}-{F}_{blank}}$$

F_test_ - fluorescence of the treated wells; F_control_ - fluorescence of the untreated wells; F_blank_- fluorescence of the unstained wells.

### Apoptosis detection

To determinutee the ability of the compounds to induce cellular apoptosis, PC-3 and DU-145 cells were plated with 5 × 10^5^ cells/well in 6 well plate. Cells were treated with compound **1** or **2** at IC_50_ concentration of each compound for 48 h. The cells were washed twice with PBS, and resuspended in 50 µl binding buffer, 2.5 ml Annexin V-FITC and 0.5 µl 7-aminuteoactinomycin D (7-AAD, labels GC-rich regions of DNA in permeabilized cells). The above mix of cells were incubated for 15 minute in the dark, followed by the addition of 200 µl binding buffer. Approximately 300 cells were analyzed by epifluorescence microscope (Nikon-Eclipse Ti-E inverted fluorescence microscope) under 20X objective for each analysis. Three biological repeats and two technical were used for each condition.

### Caspase 3/7 assay

PC-3 and DU-145 cells were plated in 96-well white plate at a density of 1 × 10^4^ cells/well 100 µl of cell culture medium. After 24 h of incubation, the cells were treated with IC_50_ concentration of the compound **1** and **2** for 5 h. Caspase 3/7 activity of cells was measured using Caspase-Glo® 3/7 Assay kit (Promega, Madison USA). Cells were equilibrated at room temperature (RT) for 30 minute. 100 µl of Caspase-Glo reagent was added to cells and incubated for 1 h at RT in dark condition. Luminuteescence of the sample was measured using Magellan^TM^ microplate reader. The fold increase of caspase 3/7 activity was calculated by applying the equation used for ROS.

### Statistical analysis

All the experiments were performed with three biological and technical repeats. The data was presented as the mean ± SEM. Statistical analysis was carried out by Student’s t-test using GraphPad Prism 7.0 software. The differences among the experimental samples were analysed with one-way ANOVA. Statistical significance was considered with the P-value of <0.05.

## Supplementary information

Supplemental file 1
