# Supplementary material for: Synthesis and preclinical validation of novel P2Y1 receptor ligands as a potent anti-prostate cancer agent
Source: Sci Rep. 2019 Dec 12;9:18938. doi: 10.1038/s41598-019-55194-8 (PMC6908675; doi:10.1038/s41598-019-55194-8)
Supplement: Supplementary file 1 — Supplemental file 1 [file 41598_2019_55194_MOESM1_ESM.pdf]

Synthesis and preclinical validation of novel P2Y1 receptor ligands as a potent anti-prostate cancer agent

Hien Thi Thu Le[1], Tatu Rimpilainen[2], Saravanan Konda Mani[3], Akshaya Murugesan[1,4], Olli Yli-Harja[5,6], Nuno R. Candeias[2] and Meenakshisundaram Kandhavelu[1]

[1]Molecular Signaling Lab, Faculty of Medicine and Health Technology, Tampere University and BioMediTech, P.O.Box 553, 33101 Tampere, Finland.

[2]Faculty of Engineering and Natural Sciences, Tampere University, Korkeakoulunkatu 8, 33101 Tampere, Finland

[3]Department of Crystallography & Biophysics, University of Madras, Guindy Campus, Chennai – 600 025, India

[4]Department of Biotechnology, Lady Doak College, Thallakulam, Madurai – 625002, India.

[5]Computational Systems Biology Research Group, Faculty of Medicine and Health Technology and BioMediTech, Tampere University, P.O.Box 553, 33101 Tampere, Finland.

[6]Institute for Systems Biology, 1441N 34th Street, Seattle, WA 98103-8904, USA

Corresponding author: meenakshisundaram.kandhavelu@tuni.fi

Additional Information

Supplementary information: Nuclear magnetic resonance (NMR) data of all the compounds tested, statistics table and glide score table.

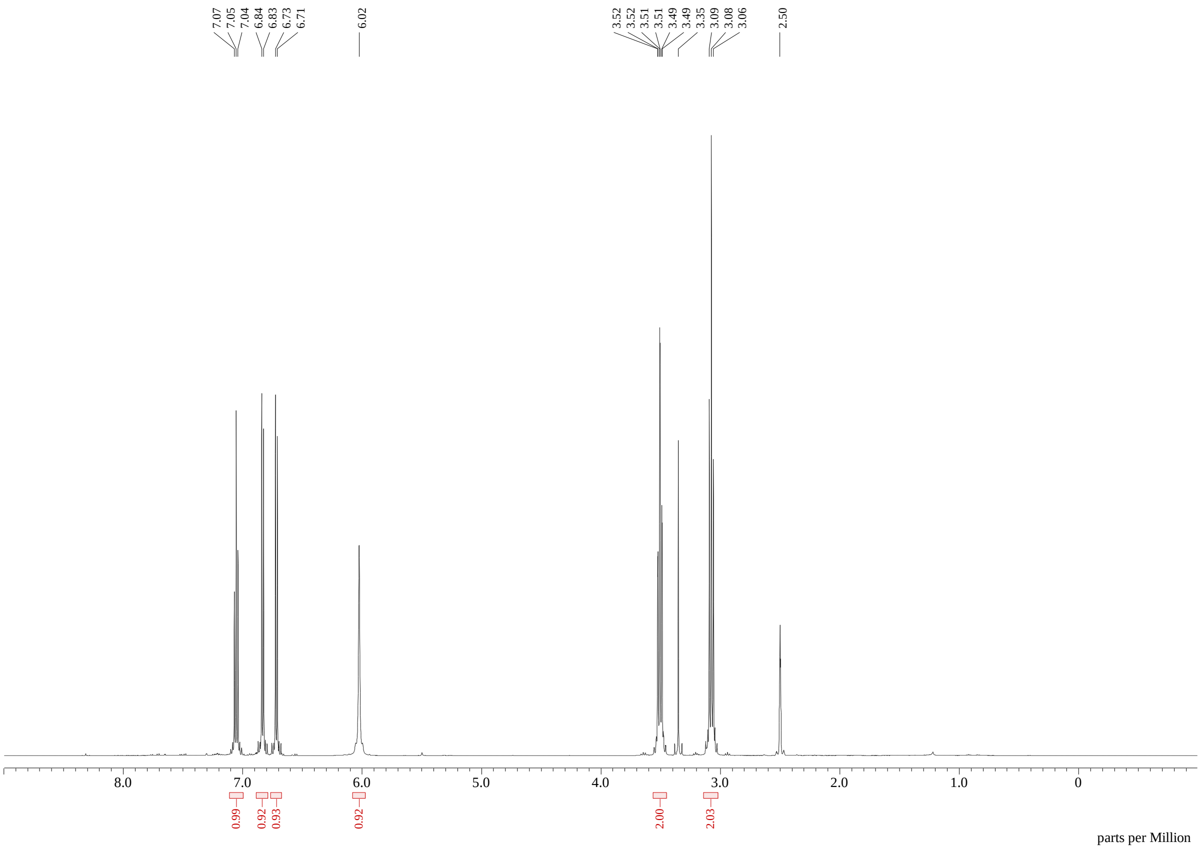

Figure S1: Indoline-4-carbonitrile ^{1}H NMR (500 MHz, DMSO-d_6)

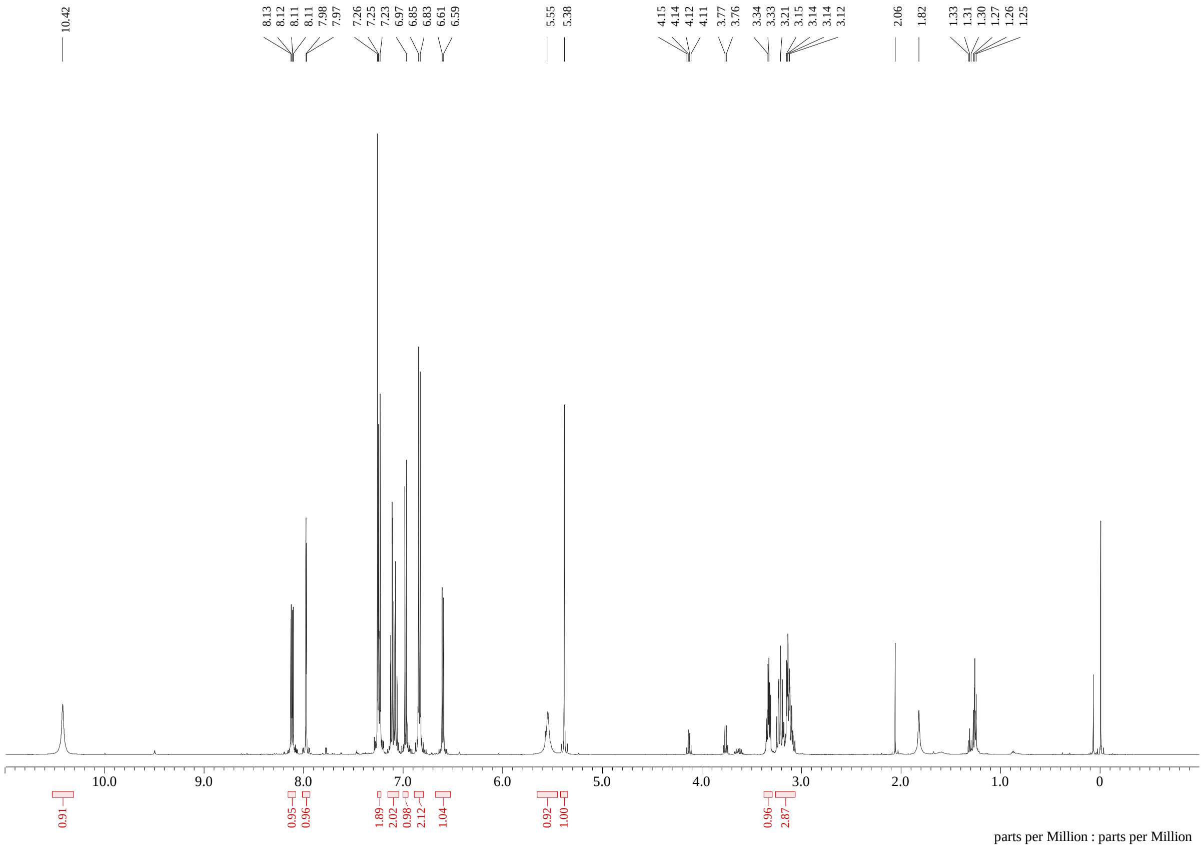

Figure S2: 1-((2-Hydroxy-5-nitrophenyl)(4-hydroxyphenyl)methyl)indoline-4-carbonitrile **(1)** ^{1}H NMR (500 MHz, $CDCl_3$)

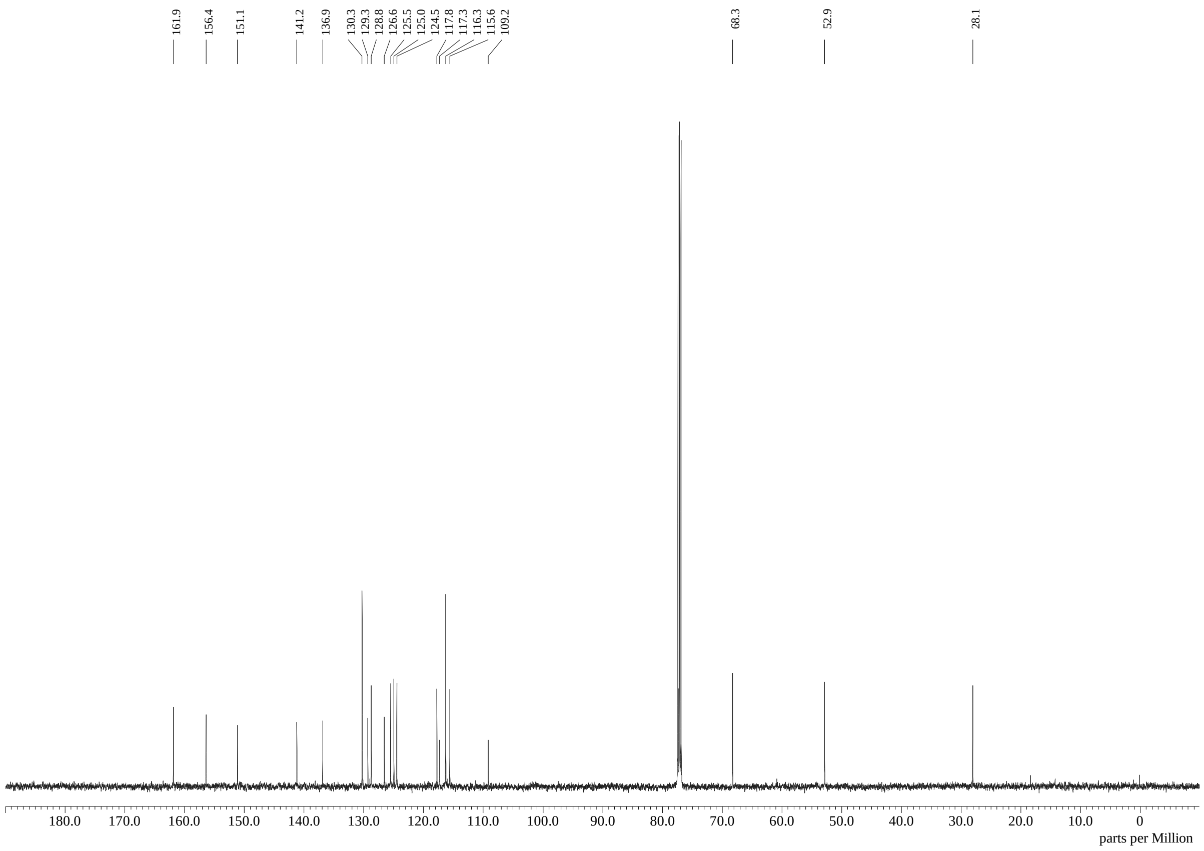

Figure S3: 1-((2-Hydroxy-5-nitrophenyl)(4-hydroxyphenyl)methyl)indoline-4-carbonitrile **(1)** ^{13}C NMR (126 MHz, $CDCl_3$)

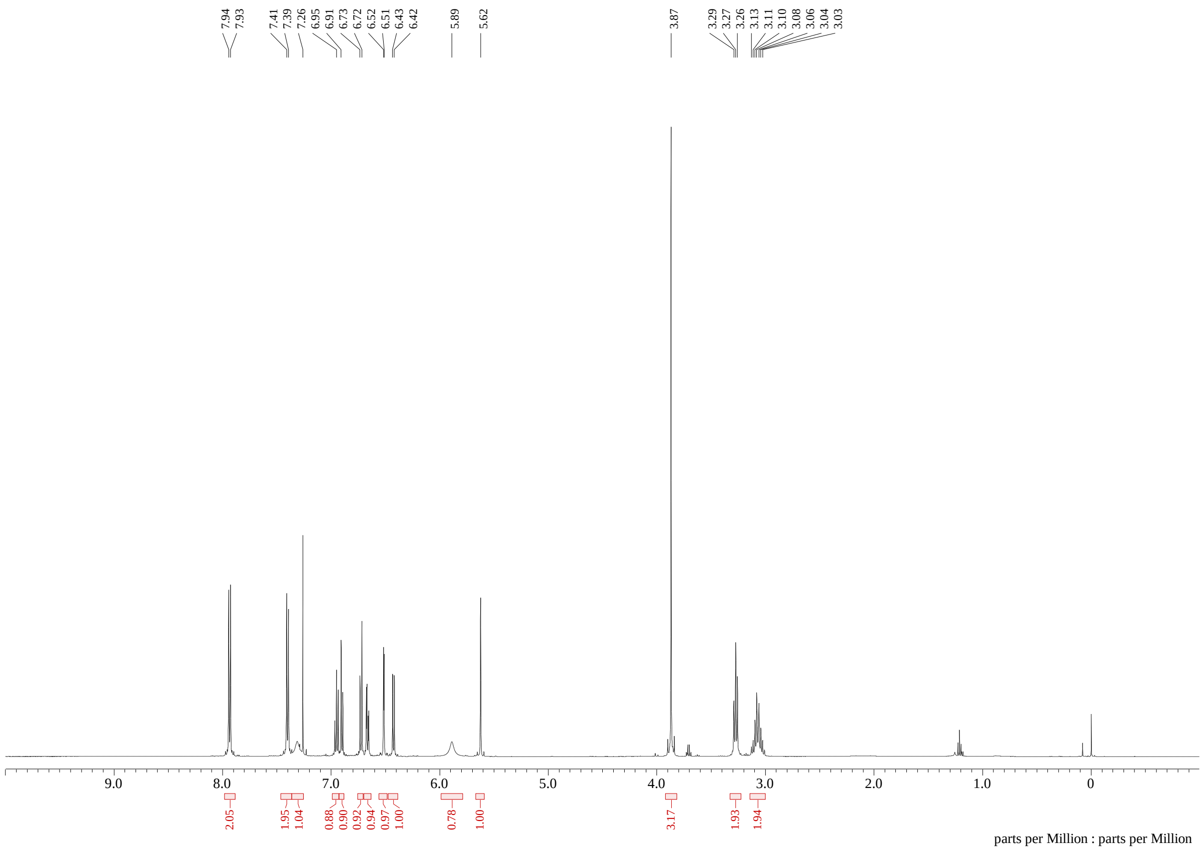

Figure S4: Methyl 4-((4-cyanoindolin-1-yl)(2,5-dihydroxyphenyl)methyl)benzoate **(2)** ^{1}H NMR (500 MHz, $CDCl_3$)

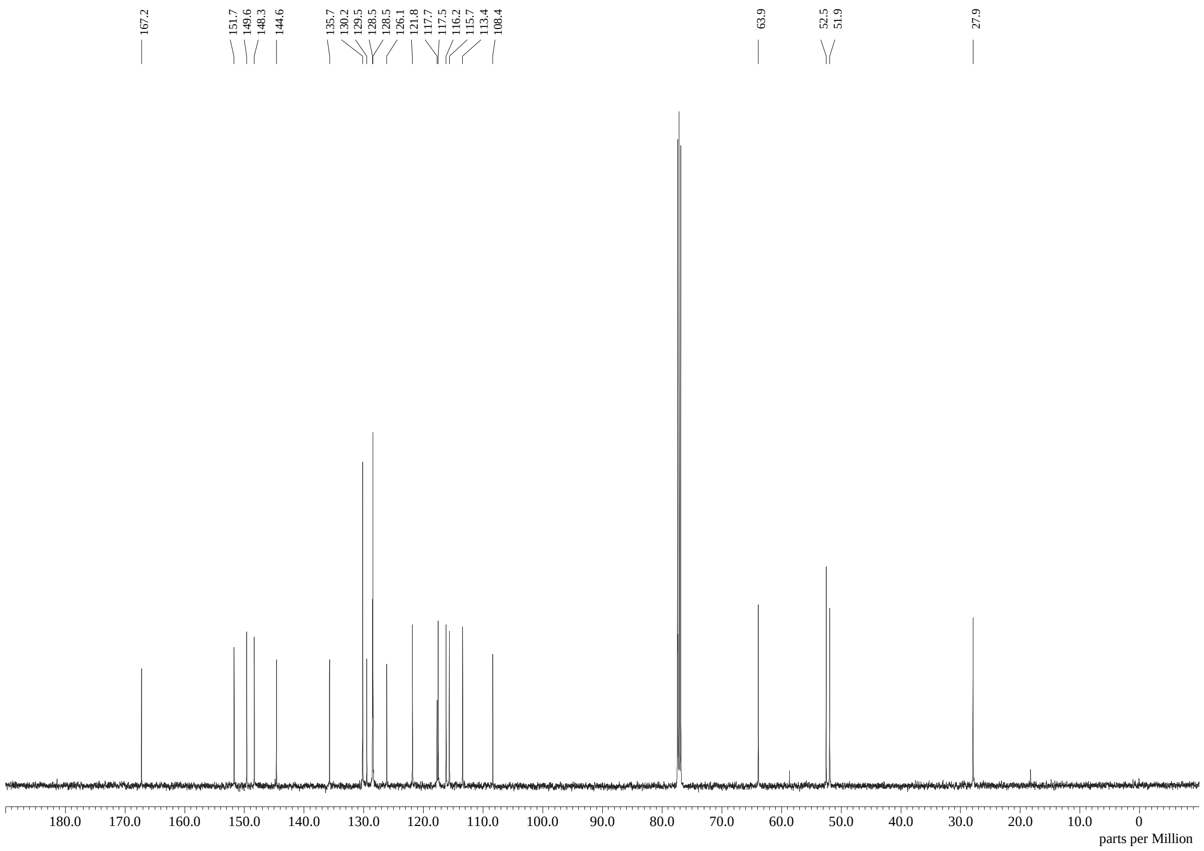

Figure S5: Methyl 4-((4-cyanoindolin-1-yl)(2,5-dihydroxyphenyl)methyl)benzoate **(2)** ^{13}C NMR (126 MHz, $CDCl_3$)

Apoptosis assay (Figure 5)			
**Sample**	**Condition**	***p* value (PC3 cells)**	***p* value (DU145 cells)**
**1**	Apoptosis	0,03619575	0,05937503
**1**	Necrosis	0,08987242	0,06505361
**2**	Apoptosis	0,0586452	0,03601299
**2**	Necrosis	0,0441798	0,04018056
MRS2365	Apoptosis	0,03852255	0,06828466
MRS2365	Necrosis	0,03030603	0,03990714

ROS assay (Figure 6A)			
**Sample**	**Condition**	***p* value (PC3 cells)**	***p* value (DU145 cells)**
**1**	16 µM	0,00279061	0,02185258
**2**	16 µM	0,03862835	0,0265219
MRS2365	2 µM	0,01159021	0,04818763

Caspase 3/7 assay (Figure 6B)			
**Sample**	**Condition**	***p* value (PC3 cells)**	***p* value (DU145 cells)**
**1**	16 µM	0,00667839	0,02933891
**2**	16 µM	0,0273775	0,03270986
MRS2365	2.5 µM	0,06238897	0,04752033

Calcium assay (Supplementary figure 1)					
**Sample**	**Condition**	***p* values** PC3 cells -/- P2Y1siRNA	***p* values** PC3 cells +/+ P2Y1siRNA	***p* values** DU145 cells -/- P2Y1siRNA	***p* values** DU145 cells +/+ P2Y1siRNA
**1**	25 µM	0,06354279	0,01309287	0,00901676	0,03947186
**2**	25 µM	0,02221272	0,07702665	0,03269552	0,01756453
MRS2365	2.5 µM	0,03631644	0,06690595	0,03163763	0,02882441

Cell cytotoxicity (Supplementary figure 2)					
**Samples**	**Condition**	***p* values** PC3 cells	***p* values** DU145 cells	***p* values** HEK 293 cells	***p* values** MEF cells
**1**	20 µM	0,02880517	0,06678169	0,01218926	0,04589988
**1**	100 µM	0,01543152	0,06028161	0,01035512	0,06673959
**2**	20 µM	0,01768196	0,05175247	0,01358867	0,04678087
**2**	100 µM	0,04366113	0,04913159	0,03810687	0,07463815
MRS2365	2.5 µM	0,05574526	0,03219407	0,01673959	0,02479666

Table S1: *p* value based on ANOVA test.

Title	Formula	glide gscore
426	C22H17N3O4	-7.39
636	C24H20N2O4	-6.93
434	C22H20ClNO3	-6.89
362	C23H22N2O5	-6.89
655	C25H25NO4	-6.86
422	C22H20N2O5	-6.83
447	C21H17BrClNO2	-6.78
466	C21H17BrClNO2	-6.76
423	C21H17ClN2O4	-6.69
62	C23H22N2O4S	-6.66
471	C21H17BrClNO2	-6.61
726	C23H19N3O3	-6.60
481	C23H20N2O3	-6.59
638	C25H25NO5	-6.56
436	C21H17BrClNO2	-6.55
361	C22H20N2O4	-6.55
465	C21H17Cl2NO2	-6.55
606	C24H19N3O5	-6.54
421	C21H18N2O4	-6.53
470	C22H20BrNO3	-6.52
448	C21H17Br2NO2	-6.51
474	C22H17BrN2O2	-6.49
542	C28H24N2O4	-6.47
433	C21H18ClNO2	-6.47
614	C24H22ClNO4	-6.46
456	C22H18N2O3	-6.45
601	C23H20N2O5	-6.45
603	C23H19ClN2O5	-6.44
366	C23H19N3O4	-6.43
604	C23H19BrN2O5	-6.42
473	C21H17BrN2O4	-6.40
467	C21H17ClN2O4	-6.37
612	C25H22N2O3	-6.36
132	C24H20N2O3	-6.35
186	C22H16ClN3O3	-6.35
440	C26H29NO3	-6.35
656	C26H27NO5	-6.34
545	C27H21N3O5	-6.33
425	C21H17N3O6	-6.32
602	C24H22N2O6	-6.31
633	C23H20ClNO4	-6.29
460	C22H20BrNO3	-6.28

665	C25H25N3O5	-6.27
472	C21H17Br2NO2	-6.27
463	C21H18ClNO2	-6.25
301	C22H17F3N2O3	-6.25
721	C22H20N2O3	-6.24
541	C27H22N2O3	-6.24
644	C24H22ClNO4	-6.23
445	C21H18BrNO2	-6.23
643	C23H20ClNO3	-6.22
184	C21H16BrClN2O3	-6.22
126	C22H16FN3O3	-6.21
626	C24H22BrNO4	-6.19
662	C26H28N2O4	-6.19
5	C22H19N3O5	-6.18
356	C25H24F3NO3	-6.18
621	C27H28ClNO3	-6.15
459	C22H20ClNO3	-6.14
122	C22H19FN2O4	-6.13
546	C28H21N3O3	-6.13
306	C23H16F3N3O3	-6.13
664	C25H25BrN2O3	-6.12
303	C22H16ClF3N2O3	-6.11
480	C24H22N2O3	-6.10
574	C27H22BrNO2	-6.10
663	C25H25ClN2O3	-6.09
723	C22H19ClN2O3	-6.08
245	C21H16BrN3O5	-6.05
457	C22H21NO3	-6.04
543	C27H21ClN2O3	-6.02
449	C21H17BrN2O4	-5.99
620	C28H31NO4	-5.98
645	C23H19Cl2NO3	-5.98
304	C22H16BrF3N2O3	-5.95
66	C23H19N3O3S	-5.94
435	C21H17Cl2NO2	-5.93
125	C21H16FN3O5	-5.91
635	C23H20N2O6	-5.90
244	C21H16Br2N2O3	-5.90
652	C23H19Br2NO3	-5.89
544	C27H21BrN2O3	-5.88
469	C21H18BrNO2	-5.87
642	C25H22N2O4	-5.87
1	C22H20N2O3	-5.87

464	C22H20ClNO3	-5.86
476	C24H25NO4	-5.84
183	C21H16Cl2N2O3	-5.82
619	C27H29NO3	-5.82
572	C28H25NO3	-5.79
661	C25H26N2O3	-5.79
450	C22H17BrN2O2	-5.79
648	C24H19ClN2O3	-5.78
2	C23H22N2O4	-5.78
651	C23H19BrClNO3	-5.77
516	C24H20N2O2	-5.76
582	C29H24N2O2	-5.75
242	C22H19BrN2O4	-5.72
724	C22H19BrN2O3	-5.72
482	C24H22N2O4	-5.71
613	C23H20ClNO3	-5.68
615	C23H19Cl2NO3	-5.67
123	C21H16ClFN2O3	-5.66
458	C23H23NO4	-5.66
485	C23H19N3O5	-5.65
624	C28H28N2O3	-5.64
363	C22H19ClN2O4	-5.64
453	C21H18ClNO3	-5.63
454	C21H18BrNO3	-5.63
658	C25H24BrNO4	-5.62
722	C23H22N2O4	-5.61
6	C23H19N3O3	-5.61
596	C30H29NO3	-5.61
483	C23H19ClN2O3	-5.60
124	C21H16BrFN2O3	-5.59
439	C25H27NO2	-5.58
478	C23H22BrNO3	-5.57
38	C22H21NO3	-5.56
452	C22H21NO4	-5.55
121	C21H17FN2O3	-5.54
555	C27H21Cl2NO	-5.54
716	C28H33NO3	-5.52
484	C23H19BrN2O3	-5.51
468	C22H17ClN2O2	-5.51
66	C24H22N2O2	-5.50
618	C24H19ClN2O3	-5.50
364	C22H19BrN2O4	-5.50
571	C27H23NO2	-5.48

443	C25H26N2O4	-5.48
243	C21H16BrClN2O3	-5.45
573	C27H22ClNO2	-5.45
451	C21H19NO3	-5.43
4	C22H19BrN2O3	-5.42
780	C25H24N2O2	-5.41
241	C21H17BrN2O3	-5.41
64	C22H19BrN2O3S	-5.40
755	C22H20N2O4	-5.40
575	C27H22N2O4	-5.39
586	C27H21BrClNO	-5.38
446	C22H20BrNO3	-5.38
632	C24H23NO5	-5.38
512	C24H23NO3	-5.37
595	C29H27NO2	-5.37
61	C22H20N2O3S	-5.37
666	C26H25N3O3	-5.35
486	C24H19N3O3	-5.35
566	C28H24BrNO2	-5.35
444	C26H26N2O2	-5.35
60	C25H24N2O2	-5.34
438	C22H17ClN2O2	-5.34
660	C26H24N2O4	-5.32
576	C28H22N2O2	-5.29
246	C22H16BrN3O3	-5.29
477	C23H22ClNO3	-5.29
725	C22H19N3O5	-5.29
302	C23H19F3N2O4	-5.28
417	C24H24ClNO3	-5.27
428	C23H23NO3	-5.26
185	C21H16ClN3O5	-5.26
182	C22H19ClN2O4	-5.26
177	C23H21ClFNO2	-5.26
432	C23H20N2O2	-5.26
393	C22H20ClNO3	-5.25
641	C24H22N2O6	-5.25
26	C23H22BrNO2	-5.25
579	C28H24ClNO2	-5.24
360	C25H21F3N2O2	-5.24
622	C27H28BrNO3	-5.23
475	C23H23NO3	-5.22
625	C23H20BrNO3	-5.20
116	C25H27NO3S	-5.20

578	C29H27NO3	-5.20
492	C25H22N2O	-5.20
105	C22H20ClNO2	-5.20
631	C23H21NO4	-5.20
429	C22H20ClNO2	-5.19
357	C24H21ClF3NO2	-5.18
590	C28H24BrNO2	-5.17
600	C30H26N2O2	-5.17
305	C22H16F3N3O5	-5.17
36	C23H20N2O2	-5.16
650	C24H22BrNO4	-5.16
358	C24H21BrF3NO2	-5.16
181	C21H17ClN2O3	-5.15
778	C24H24BrNO2	-5.15
637	C24H23NO4	-5.14
605	C23H19N3O7	-5.14
106	C22H20BrNO2	-5.13
237	C23H21Cl2NO2	-5.13
176	C24H24FNO3	-5.13
396	C23H20N2O3	-5.13
420	C25H24N2O3	-5.13
375	C22H19Cl2NO2	-5.12
629	C23H19BrN2O5	-5.12
588	C28H21ClN2O	-5.11
654	C24H19BrN2O3	-5.11
583	C27H22ClNO	-5.11
178	C23H21BrFNO2	-5.11
365	C22H19N3O6	-5.11
430	C22H20BrNO2	-5.11
554	C28H24ClNO2	-5.11
560	C32H33NO2	-5.10
24	C22H17ClN2O	-5.10
14	C23H22ClNO2	-5.08
577	C28H25NO2	-5.08
370	C23H22BrNO2	-5.08
326	C23H19BrF3NO2	-5.08
611	C24H22N2O5	-5.07
395	C22H20N2O5	-5.07
342	C24H19F3N2O2	-5.06
584	C28H24ClNO2	-5.06
598	C29H26BrNO2	-5.06
617	C23H19ClN2O5	-5.06
462	C23H20N2O3	-5.05

597	C29H26ClNO2	-5.05
340	C23H19BrF3NO2	-5.05
236	C24H24ClNO3	-5.05
441	C25H26ClNO2	-5.04
179	C23H21FN2O4	-5.04
563	C31H30N2O3	-5.03
387	C22H19BrClNO2	-5.03
510	C24H19BrN2O	-5.03
738	C23H19ClN2O	-5.03
388	C22H19Br2NO2	-5.03
298	C23H21Br2NO2	-5.03
418	C24H24BrNO3	-5.03
376	C22H19BrClNO2	-5.02
548	C29H27NO2	-5.02
427	C22H21NO2	-5.01
594	C28H21BrN2O	-5.01
119	C24H24N2O4S	-5.01
32	C22H20BrNO2	-5.00
238	C23H21BrClNO2	-5.00
371	C23H22N2O4	-5.00
117	C24H24ClNO2S	-5.00
194	C22H19Cl2NO2	-5.00
536	C26H27NO3	-5.00
402	C24H22N2O3	-4.99
332	C23H20F3NO3	-4.98
416	C25H27NO4	-4.98
104	C23H23NO3	-4.98
522	C25H22N2O2	-4.97
33	C22H20ClNO2	-4.97
8	C24H25NO2	-4.97
118	C24H24BrNO2S	-4.97
593	C27H21BrN2O3	-4.96
608	C25H25NO4	-4.96
314	C23H19ClF3NO2	-4.96
479	C23H22N2O5	-4.96
56	C25H27NO3	-4.95
2	C22H21NO2	-4.95
415	C24H25NO3	-4.94
180	C24H21FN2O2	-4.94
156	C22H17FN2O2	-4.94
120	C25H24N2O2S	-4.93
239	C23H21ClN2O4	-4.93
768	C23H19ClN2O	-4.93

113	C21H18N2O4	-4.92
297	C23H21BrClNO2	-4.92
558	C28H21ClN2O	-4.91
570	C28H21BrN2O	-4.91
348	C23H16ClF3N2O	-4.91
187	C22H20ClNO	-4.91
58	C24H24BrNO2	-4.90
562	C31H30BrNO	-4.90
760	C23H22BrNO2	-4.89
411	C22H19BrClNO2	-4.89
514	C23H20BrNO2	-4.89
30	C23H19BrN2O	-4.89
300	C24H21BrN2O2	-4.89
57	C24H24ClNO2	-4.89
587	C27H21ClN2O3	-4.88
647	C23H19ClN2O5	-4.88
609	C24H22ClNO3	-4.88
580	C28H24BrNO2	-4.87
70	C23H22BrNOS	-4.87
164	C22H19ClFNO2	-4.87
331	C22H18F3NO2	-4.87
547	C28H25NO	-4.87
599	C29H26N2O4	-4.87
336	C23H17F3N2O2	-4.87
359	C24H21F3N2O4	-4.86
377	C22H19ClN2O4	-4.86
585	C27H21Cl2NO	-4.85
271	C21H18BrNO2	-4.85
50	C23H22BrNO2	-4.84
324	C27H25F3N2O	-4.84
34	C22H20BrNO2	-4.83
623	C27H28N2O5	-4.83
152	C22H20FNO3	-4.83
690	C26H25BrN2O	-4.83
42	C22H18N2O2	-4.83
455	C21H18N2O5	-4.82
93	C22H20ClNO2S	-4.82
111	C22H19BrClNOS	-4.82
63	C22H19ClN2O3S	-4.82
86	C22H20ClNO2	-4.82
65	C22H19N3O5S	-4.82
639	C24H22ClNO4	-4.81
559	C31H31NO	-4.81

240	$C_{24}H_{21}ClN_2O_2$	-4.80
260	$C_{26}H_{28}BrNO_2$	-4.78
437	$C_{21}H_{17}ClN_2O_4$	-4.78
646	$C_{23}H_{19}BrClNO_3$	-4.78
630	$C_{24}H_{19}BrN_2O_3$	-4.78
392	$C_{23}H_{23}NO_4$	-4.78
412	$C_{22}H_{19}Br_2NO_2$	-4.78
216	$C_{22}H_{17}ClN_2O_2$	-4.77
384	$C_{27}H_{28}N_2O_2$	-4.77
234	$C_{22}H_{16}BrClN_2O$	-4.77
569	$C_{27}H_{21}BrN_2O_3$	-4.77
102	$C_{24}H_{22}N_2O_2S$	-4.76
153	$C_{21}H_{17}ClFNO_2$	-4.76
159	$C_{22}H_{19}ClFNO_2$	-4.76
341	$C_{23}H_{19}F_3N_2O_4$	-4.76
200	$C_{26}H_{28}ClNO_2$	-4.76
405	$C_{22}H_{19}Cl_2NO_2$	-4.76
389	$C_{22}H_{19}BrN_2O_4$	-4.75
607	$C_{24}H_{23}NO_3$	-4.75
162	$C_{23}H_{19}FN_2O_2$	-4.75
758	$C_{24}H_{25}NO_3$	-4.75
535	$C_{25}H_{25}NO_2$	-4.74
657	$C_{25}H_{24}ClNO_4$	-4.74
552	$C_{29}H_{24}N_2O$	-4.74
151	$C_{21}H_{18}FNO_2$	-4.74
192	$C_{23}H_{19}ClN_2O$	-4.74
762	$C_{24}H_{22}N_2O_2$	-4.73
39	$C_{21}H_{18}ClNO_2$	-4.73
121	$C_{27}H_{23}NO$	-4.73
591	$C_{27}H_{21}BrClNO$	-4.73
323	$C_{26}H_{25}F_3N_2O_3$	-4.73
31	$C_{22}H_{21}NO_2$	-4.73
42	$C_{24}H_{22}N_2O_2$	-4.73
224	$C_{22}H_{19}Cl_2NO_2$	-4.72
296	$C_{24}H_{24}BrNO_3$	-4.72
72	$C_{24}H_{22}N_2OS$	-4.72
320	$C_{27}H_{28}F_3NO_2$	-4.72
355	$C_{24}H_{22}F_3NO_2$	-4.72
759	$C_{23}H_{22}ClNO_2$	-4.71
48	$C_{23}H_{20}N_2O_2$	-4.71
62	$C_{24}H_{25}NO_3$	-4.71
299	$C_{23}H_{21}BrN_2O_4$	-4.70
158	$C_{23}H_{22}FNO_3$	-4.69

206	C22H19BrClNO2	-4.69
254	C22H19BrClNO2	-4.69
754	C22H20BrNO2	-4.69
235	C23H22ClNO2	-4.69
511	C23H21NO2	-4.68
431	C22H20N2O4	-4.67
564	C32H30N2O	-4.67
696	C26H26N2O2	-4.67
518	C25H25NO3	-4.66
48	C23H19ClN2O	-4.66
211	C21H18ClNO2	-4.66
214	C21H17BrClNO2	-4.66
124	C27H22BrNO	-4.66
160	C22H19BrFNO2	-4.66
693	C25H26ClNO2	-4.66
140	C26H28FNO2	-4.66
20	C22H20ClNO2	-4.65
40	C23H22BrNO2	-4.64
96	C23H20N2O2S	-4.64
499	C27H29NO	-4.63
126	C28H22N2O	-4.63
494	C24H22ClNO2	-4.62
123	C27H22ClNO	-4.62
32	C23H23NO3	-4.62
534	C24H19BrN2O	-4.61
610	C24H22BrNO3	-4.61
312	C24H19F3N2O	-4.61
513	C23H20ClNO2	-4.61
222	C23H19ClN2O2	-4.61
568	C27H21Br2NO	-4.61
40	C21H18BrNO2	-4.61
212	C22H20ClNO3	-4.61
195	C21H16Cl3NO	-4.60
353	C22H16BrF3N2O3	-4.60
6	C22H18N2O	-4.60
487	C24H23NO	-4.60
506	C24H22BrNO2	-4.60
761	C23H22N2O4	-4.59
549	C28H24ClNO	-4.59
281	C22H19BrN2O4	-4.59
528	C24H19ClN2O	-4.58
329	C22H16BrF3N2O3	-4.57
114	C23H19BrN2OS	-4.57

517	C24H23NO2	-4.56
556	C27H21BrClNO	-4.56
198	C22H16Cl2N2O	-4.56
168	C22H16ClFN2O	-4.56
561	C31H30ClNO	-4.56
295	C23H22BrNO2	-4.55
54	C22H17ClN2O	-4.55
634	C23H20BrNO4	-4.55
78	C23H20N2OS	-4.55
313	C22H17ClF3NO	-4.54
557	C27H21ClN2O3	-4.54
110	C22H21NO3	-4.54
775	C24H25NO2	-4.54
407	C22H19ClN2O4	-4.54
44	C23H22ClNO2	-4.54
36	C22H17BrN2O	-4.54
398	C24H25NO4	-4.54
408	C23H19ClN2O2	-4.54
419	C24H24N2O5	-4.54
592	C27H21Br2NO	-4.54
230	C22H19BrClNO2	-4.53
272	C22H20BrNO3	-4.53
372	C24H22N2O2	-4.53
228	C22H16Cl2N2O	-4.53
317	C22H16ClF3N2O3	-4.52
500	C28H31NO2	-4.52
273	C21H17BrClNO2	-4.52
767	C22H19ClN2O3	-4.52
140	C23H23NO2	-4.52
691	C25H27NO2	-4.52
94	C22H20BrNO2S	-4.51
91	C22H21NO2S	-4.51
68	C24H25NO2S	-4.51
154	C21H17BrFNO2	-4.50
719	C27H30N2O4	-4.50
756	C23H20N2O2	-4.49
347	C22H16ClF3N2O3	-4.49
282	C23H19BrN2O2	-4.48
3	C22H19ClN2O3	-4.48
84	C22H17FN2O	-4.48
249	C22H19BrClNO	-4.47
203	C25H25ClN2O3	-4.47
319	C26H26F3NO	-4.47

276	C22H17BrN2O2	-4.46
290	C22H19Br2NO2	-4.46
90	C22H17ClN2O	-4.46
138	C22H16ClFN2O	-4.45
125	C27H22N2O3	-4.44
278	C23H22BrNO3	-4.43
333	C22H17ClF3NO2	-4.43
692	C26H29NO3	-4.43
37	C21H19NO2	-4.43
114	C22H18N2O2	-4.43
188	C23H22ClNO2	-4.42
87	C21H17Cl2NO	-4.42
135	C21H16Cl2FNO	-4.42
325	C22H17BrF3NO	-4.42
504	C28H28N2O	-4.42
105	C22H19Cl2NOS	-4.41
82	C21H17BrFNO	-4.41
155	C21H17FN2O4	-4.40
330	C23H16BrF3N2O	-4.40
442	C25H26BrNO2	-4.40
488	C25H25NO2	-4.40
702	C27H28N2O2	-4.40
345	C22H16Cl2F3NO	-4.40
750	C23H19BrN2O	-4.40
102	C23H17F3N2O	-4.39
414	C23H19BrN2O2	-4.39
707	C25H25ClN2O3	-4.39
280	C22H19Br2NO2	-4.39
335	C22H17F3N2O4	-4.39
78	C23H19ClN2OS	-4.39
404	C23H22ClNO3	-4.38
174	C22H16BrFN2O	-4.38
540	C26H24N2O2	-4.38
581	C28H24N2O4	-4.37
294	C22H16Br2N2O	-4.37
215	C21H17ClN2O4	-4.36
728	C24H25NO2	-4.36
343	C22H17ClF3NO	-4.35
715	C27H31NO2	-4.35
747	C22H19BrClNO	-4.35
132	C23H19FN2O	-4.35
368	C24H25NO3	-4.35
131	C22H19FN2O3	-4.35

288	C22H16BrClN2O	-4.34
113	C22H19BrN2O3S	-4.34
258	C22H16BrClN2O	-4.34
131	C23H20N2O5	-4.34
567	C27H21BrClNO	-4.34
275	C21H17BrN2O4	-4.34
308	C24H22F3NO2	-4.34
130	C22H19BrFNO	-4.34
84	C27H28N2OS	-4.34
344	C23H19ClF3NO2	-4.34
213	C21H17Cl2NO2	-4.33
394	C22H20BrNO3	-4.33
143	C25H25FN2O3	-4.33
753	C22H20ClNO2	-4.33
776	C25H27NO3	-4.33
628	C23H19Br2NO3	-4.33
97	C22H18F3NO	-4.33
321	C26H25ClF3NO	-4.32
264	C26H25BrN2O	-4.32
92	C22H20BrNO2	-4.32
270	C22H16Br2N2O	-4.32
369	C23H22ClNO2	-4.32
322	C26H25BrF3NO	-4.31
490	C24H22BrNO	-4.31
12	C24H22N2O	-4.30
680	C30H37NO2	-4.30
147	C21H16BrClFNO	-4.30
277	C22H20BrNO2	-4.30
9	C21H17ClN2O3	-4.30
170	C22H19BrFNO2	-4.30
515	C23H20N2O4	-4.30
175	C23H22FNO2	-4.29
713	C25H25BrN2O3	-4.28
150	C22H16BrFN2O	-4.28
496	C23H19BrClNO	-4.28
352	C22H16Br2F3NO	-4.27
100	C22H17BrF3NO	-4.27
136	C21H16BrClFNO	-4.27
141	C25H25ClFNO	-4.27
10	C23H22BrNO	-4.27
349	C22H17BrF3NO	-4.27
128	C23H22FNO2	-4.26
386	C23H22BrNO3	-4.26

3	C21H18ClNO	-4.26
22	C21H17BrClNO	-4.26
97	C23H23NO2S	-4.25
204	C26H25ClN2O	-4.25
338	C24H22F3NO3	-4.25
93	C21H17BrClNO	-4.24
773	C22H19BrN2O3	-4.24
616	C23H19BrClNO3	-4.24
88	C21H17BrClNO	-4.24
315	C22H16Cl2F3NO	-4.23
501	C27H28ClNO	-4.23
740	C27H31NO2	-4.23
379	C26H29NO2	-4.23
171	C21H16BrClFNO	-4.23
268	C21H16Br3NO	-4.23
694	C25H26BrNO2	-4.23
565	C27H22BrNO	-4.23
659	C25H24N2O6	-4.23
18	C23H19ClN2O	-4.22
461	C22H20N2O5	-4.22
328	C22H16Br2F3NO	-4.22
106	C22H19BrClNOS	-4.22
208	C21H16Br2ClNO	-4.21
752	C23H23NO3	-4.21
74	C23H23NO2S	-4.21
92	C23H23NO3S	-4.21
259	C25H26BrNO	-4.21
4	C21H18BrNO	-4.21
12	C22H17N3O3	-4.21
309	C23H19ClF3NO	-4.21
503	C27H28N2O3	-4.21
21	C26H28ClNO	-4.21
550	C28H24BrNO	-4.21
649	C23H20BrNO3	-4.20
318	C23H16ClF3N2O	-4.20
23	C26H28N2O3	-4.20
736	C22H19BrClNO	-4.20
383	C26H28N2O4	-4.19
263	C25H25BrN2O3	-4.18
108	C23H19ClN2OS	-4.18
166	C21H16BrClFNO	-4.18
553	C27H22ClNO	-4.18
90	C23H19BrN2OS	-4.17

196	C21H16BrCl2NO	-4.17
15	C22H19Cl2NO	-4.17
24	C27H28N2O	-4.17
334	C22H17BrF3NO2	-4.17
676	C25H25BrClNO	-4.17
14	C23H23NO2	-4.17
110	C23H22BrNO2S	-4.17
779	C24H24N2O4	-4.16
115	C24H25NO2S	-4.16
59	C24H24N2O4	-4.16
627	C23H19BrClNO3	-4.16
381	C26H28ClNO2	-4.16
15	C22H20ClNO	-4.15
107	C22H19ClN2O3S	-4.15
262	C25H25Br2NO	-4.15
256	C21H16Br2ClNO	-4.14
45	C22H19Cl2NO	-4.14
653	C23H19BrN2O5	-4.14
279	C22H19BrClNO2	-4.13
551	C28H24N2O3	-4.13
77	C22H19ClN2O3S	-4.13
774	C23H19BrN2O	-4.13
210	C22H16BrClN2O	-4.13
399	C23H22ClNO3	-4.13
316	C22H16BrClF3NO	-4.13
21	C21H17Cl2NO	-4.13
80	C22H20FNO2	-4.13
52	C22H19Br2NO	-4.12
538	C25H24BrNO2	-4.12
191	C22H19ClN2O3	-4.12
390	C23H19BrN2O2	-4.12
735	C22H19Cl2NO	-4.12
489	C24H22ClNO	-4.12
253	C21H17BrClNO	-4.11
25	C22H20BrNO	-4.11
232	C21H16Br2ClNO	-4.11
274	C21H17Br2NO2	-4.10
686	C26H28BrNO2	-4.10
746	C23H22BrNO2	-4.10
16	C22H20BrNO	-4.10
391	C22H21NO3	-4.10
730	C23H22BrNO	-4.10
219	C22H19Cl2NO2	-4.09

202	C25H25BrClNO	-4.09
250	C22H19Br2NO	-4.09
20	C27H31NO2	-4.09
22	C26H28BrNO	-4.09
218	C23H22ClNO3	-4.08
128	C24H23NO4	-4.08
255	C21H16BrCl2NO	-4.08
197	C21H16Cl2N2O3	-4.08
777	C24H24ClNO2	-4.08
83	C21H17FN2O3	-4.08
201	C25H25Cl2NO	-4.08
225	C21H16Cl3NO	-4.08
142	C22H20BrNO	-4.08
29	C22H19BrN2O3	-4.08
382	C26H28BrNO2	-4.07
257	C21H16BrClN2O3	-4.07
765	C22H19Cl2NO	-4.06
46	C22H19BrClNO	-4.06
11	C23H22N2O3	-4.05
89	C22H19BrN2O3S	-4.05
742	C26H28BrNO	-4.05
10	C21H17BrN2O3	-4.05
672	C27H28N2O	-4.05
95	C22H20N2O4S	-4.05
33	C21H17BrClNO	-4.05
142	C25H25BrFNO	-4.04
157	C22H20FNO2	-4.04
28	C25H26BrNO	-4.04
532	C23H19Br2NO	-4.04
502	C27H28BrNO	-4.03
521	C24H22N2O4	-4.03
165	C21H16Cl2FNO	-4.03
751	C22H21NO2	-4.03
764	C23H22ClNO2	-4.03
537	C25H24ClNO2	-4.02
64	C23H22BrNO2	-4.02
346	C22H16BrClF3NO	-4.02
35	C22H20N2O4	-4.02
100	C23H22BrNO2S	-4.02
74	C23H22ClNO2S	-4.01
146	C22H19BrFNO2	-4.01
351	C22H16BrClF3NO	-4.01
744	C27H28N2O	-4.01

137	C21H16ClFN2O3	-4.01
727	C23H23NO	-4.01
112	C22H19Br2NOS	-4.00
354	C23H16BrF3N2O	-4.00
509	C23H19BrN2O3	-4.00
286	C21H16Br2ClNO	-3.99
731	C23H22N2O3	-3.99
741	C26H28ClNO	-3.99
743	C26H28N2O3	-3.99
7	C23H23NO	-3.98
56	C22H20BrNO2	-3.98
144	C26H25FN2O	-3.98
284	C22H19BrClNO2	-3.97
495	C23H19Cl2NO	-3.97
51	C21H17Cl2NO	-3.97
729	C23H22ClNO	-3.97
252	C23H19BrN2O	-3.97
497	C23H19ClN2O3	-3.96
173	C21H16BrFN2O3	-3.96
327	C22H16BrClF3NO	-3.96
34	C21H17Br2NO	-3.96
47	C22H19ClN2O3	-3.96
96	C22H17BrN2O	-3.95
378	C23H19ClN2O2	-3.95
59	C21H17BrN2O3	-3.94
248	C23H22BrNO2	-3.94
668	C27H31NO2	-3.94
73	C22H20ClNOS	-3.94
337	C23H20F3NO2	-3.93
539	C25H24N2O4	-3.93
88	C22H19Br2NOS	-3.93
41	C21H18N2O4	-3.93
261	C25H25BrClNO	-3.93
640	C24H22BrNO4	-3.93
734	C23H22ClNO2	-3.93
339	C23H19ClF3NO2	-3.92
251	C22H19BrN2O3	-3.92
209	C21H16BrClN2O3	-3.92
698	C27H31NO3	-3.92
172	C21H16Br2FNO	-3.92
30	C26H26N2O	-3.92
732	C24H22N2O	-3.91
703	C25H26ClNO	-3.91

307	C23H20F3NO	-3.91
350	C23H19BrF3NO2	-3.91
65	C23H22N2O4	-3.91
226	C21H16BrCl2NO	-3.91
491	C24H22N2O3	-3.91
116	C24H23NO2	-3.91
60	C22H17BrN2O	-3.90
68	C23H23NO2	-3.90
108	C23H20N2O2	-3.90
127	C22H20FNO	-3.90
53	C21H17ClN2O3	-3.90
43	C22H20ClNO	-3.90
149	C21H16BrFN2O3	-3.90
708	C26H25ClN2O	-3.90
367	C23H23NO2	-3.89
589	C27H22BrNO	-3.89
41	C23H22N2O4	-3.89
217	C22H20ClNO2	-3.89
148	C21H16Br2FNO	-3.89
221	C22H19ClN2O4	-3.89
223	C21H17Cl2NO	-3.89
233	C21H16BrClN2O3	-3.89
85	C22H20BrNOS	-3.88
667	C26H29NO	-3.88
705	C25H25Cl2NO	-3.88
704	C26H28ClNO2	-3.87
13	C22H20ClNO	-3.87
134	C22H19ClFNO2	-3.86
35	C21H17BrN2O3	-3.86
67	C23H23NOS	-3.86
401	C23H22N2O5	-3.86
291	C21H16Br2ClNO	-3.86
706	C25H25BrClNO	-3.85
695	C25H26N2O4	-3.85
80	C27H31NO2S	-3.85
247	C22H20BrNO	-3.84
103	C22H20ClNOS	-3.84
129	C22H19ClFNO	-3.83
52	C21H17BrClNO	-3.83
681	C29H34ClNO	-3.83
122	C28H25NO2	-3.82
711	C25H25BrClNO	-3.82
63	C23H22ClNO2	-3.82

19	C26H29NO	-3.82
292	C21H16Br3NO	-3.82
410	C23H22BrNO3	-3.82
120	C24H20N2O	-3.82
44	C23H23NO3	-3.81
107	C22H20N2O4	-3.81
11	C21H17N3O5	-3.81
79	C26H29NOS	-3.81
285	C21H16BrCl2NO	-3.81
498	C24H19ClN2O	-3.80
523	C23H20ClNO	-3.80
141	C22H20ClNO	-3.80
699	C26H28ClNO2	-3.80
58	C21H17Br2NO	-3.80
49	C21H18ClNO	-3.79
530	C24H22BrNO2	-3.79
16	C22H19BrClNO	-3.79
51	C22H19BrClNO	-3.79
54	C23H19BrN2O	-3.78
145	C21H17BrFNO	-3.78
505	C23H20BrNO	-3.78
267	C21H16Br2ClNO	-3.78
8	C22H20N2O4	-3.78
81	C26H28ClNOS	-3.78
75	C22H19Cl2NOS	-3.78
673	C25H26ClNO	-3.77
310	C23H19BrF3NO	-3.77
766	C22H19BrClNO	-3.77
193	C21H17Cl2NO	-3.77
266	C22H19Br2NO2	-3.76
98	C23H20F3NO2	-3.76
17	C22H19ClN2O3	-3.76
169	C21H17BrFNO	-3.76
508	C23H19Br2NO	-3.75
771	C22H19BrClNO	-3.75
53	C22H19BrN2O3	-3.75
413	C22H19BrN2O4	-3.75
86	C23H22BrNO2S	-3.74
265	C21H17Br2NO	-3.74
385	C22H20BrNO2	-3.73
533	C23H19BrN2O3	-3.73
772	C22H19Br2NO	-3.73
29	C25H26N2O3	-3.73

403	C22H20ClNO2	-3.73
18	C23H20N2O	-3.73
519	C24H22ClNO2	-3.72
737	C22H19ClN2O3	-3.71
133	C21H17ClFNO	-3.71
99	C23H22ClNO2S	-3.71
28	C22H19Br2NO	-3.70
717	C27H30ClNO2	-3.70
109	C21H19NO2	-3.70
700	C26H28BrNO2	-3.70
400	C23H22BrNO3	-3.69
718	C27H30BrNO2	-3.69
757	C23H23NO2	-3.69
526	C23H19BrClNO	-3.68
207	C21H16BrCl2NO	-3.68
227	C21H16Cl2N2O3	-3.68
220	C22H19BrClNO2	-3.67
714	C26H25BrN2O	-3.67
406	C22H19BrClNO2	-3.66
520	C24H22BrNO2	-3.66
43	C22H21NO2	-3.66
720	C28H30N2O2	-3.64
101	C22H17F3N2O3	-3.64
190	C22H19BrClNO	-3.64
26	C26H29NO2	-3.64
112	C21H18BrNO2	-3.64
311	C23H19F3N2O3	-3.64
409	C22H20BrNO2	-3.63
733	C22H20ClNO	-3.63
117	C23H20ClNO	-3.63
109	C22H20BrNOS	-3.63
119	C23H20N2O3	-3.63
83	C26H28N2O3S	-3.63
143	C22H20N2O3	-3.62
682	C29H34BrNO	-3.62
167	C21H16ClFN2O3	-3.62
129	C23H20ClNO3	-3.62
701	C26H28N2O4	-3.62
27	C25H26ClNO	-3.62
679	C29H35NO	-3.62
748	C22H19Br2NO	-3.62
9	C23H22ClNO	-3.62
139	C25H26FNO	-3.61

710	C26H28BrNO2	-3.61
69	C22H20ClNO	-3.61
687	C25H25BrClNO	-3.61
130	C23H20BrNO3	-3.61
688	C25H25Br2NO	-3.61
697	C26H29NO2	-3.60
205	C21H17BrClNO	-3.60
38	C24H25NO3	-3.59
529	C23H20BrNO	-3.59
82	C26H28BrNOS	-3.59
293	C21H16Br2N2O3	-3.59
111	C21H18ClNO2	-3.59
71	C23H22N2O3S	-3.58
709	C25H26BrNO	-3.58
525	C23H19Cl2NO	-3.57
37	C23H23NO2	-3.56
524	C24H22ClNO2	-3.56
7	C21H18N2O3	-3.56
527	C23H19ClN2O3	-3.56
231	C21H16BrCl2NO	-3.55
373	C22H20ClNO2	-3.55
199	C25H26ClNO	-3.55
72	C23H20N2O	-3.54
13	C22H21NO	-3.54
55	C24H25NO2	-3.53
137	C25H26N2O3	-3.52
76	C22H19BrClNOS	-3.51
712	C25H25Br2NO	-3.51
50	C22H20ClNO2	-3.51
39	C23H22ClNO2	-3.51
493	C23H20ClNO	-3.51
674	C26H28ClNO2	-3.51
75	C22H20ClNOS	-3.50
161	C22H19FN2O4	-3.50
685	C25H26BrNO	-3.50
25	C25H27NO	-3.49
745	C22H20BrNO	-3.49
739	C26H29NO	-3.49
87	C22H19BrClNOS	-3.48
289	C21H17Br2NO	-3.47
770	C23H22BrNO2	-3.47
127	C23H21NO3	-3.47
118	C23H20BrNO	-3.47

287	C21H16BrClN2O3	-3.45
69	C23H22ClNOS	-3.44
76	C22H20BrNOS	-3.44
678	C26H25ClN2O	-3.43
189	C22H19Cl2NO	-3.43
684	C30H34N2O	-3.43
71	C22H20N2O3	-3.42
19	C21H18ClNO	-3.42
61	C23H23NO2	-3.42
103	C22H21NO2	-3.41
397	C23H23NO3	-3.41
677	C25H25ClN2O3	-3.41
134	C26H29NO2	-3.41
283	C21H17BrClNO	-3.41
23	C21H17ClN2O3	-3.40
669	C26H28ClNO	-3.40
98	C24H25NO3S	-3.40
671	C26H28N2O3	-3.40
531	C23H19BrClNO	-3.38
763	C22H20ClNO	-3.37
269	C21H16Br2N2O3	-3.37
229	C21H17BrClNO	-3.36
163	C21H17ClFNO	-3.36
67	C22H21NO	-3.35
55	C21H18BrNO	-3.33
507	C23H19BrClNO	-3.33
136	C25H26BrNO	-3.33
144	C23H20N2O	-3.33
27	C22H19BrClNO	-3.31
99	C22H17ClF3NO	-3.31
683	C29H34N2O3	-3.31
670	C26H28BrNO	-3.29
138	C26H26N2O	-3.29
49	C22H20BrNO	-3.28
57	C21H17BrClNO	-3.26
135	C25H26ClNO	-3.24
89	C21H17ClN2O3	-3.24
17	C22H20N2O3	-3.24
95	C21H17BrN2O3	-3.23
46	C22H20BrNO2	-3.23
5	C21H18N2O3	-3.22
91	C21H18BrNO	-3.20
104	C23H22ClNO2S	-3.16

101	C23H22N2O4S	-3.16
675	C25H25Cl2NO	-3.15
85	C21H18ClNO	-3.06
689	C25H25BrN2O3	-3.05
1	C21H19NO	-3.04
749	C22H19BrN2O3	-2.95
81	C21H17ClFNO	-2.91
31	C21H18BrNO	-2.88
769	C22H20BrNO	-2.85
133	C25H27NO	-2.84
70	C22H20BrNO	-2.83
380	C27H31NO3	-2.72
115	C23H21NO	-2.67
77	C22H20N2O3S	-2.61
94	C21H17Br2NO	-2.48
139	C22H21NO	-2.33
45	C22H20ClNO2	-2.20
47	C22H20N2O4	-2.19
374	C23H22ClNO3	-2.10
79	C21H18FNO	-1.96
73	C22H21NOS	-1.89

Table S2: Glide gscores of 923 compounds.
